# Early life stress induces irritable bowel syndrome from childhood to adulthood in mice

**DOI:** 10.3389/fmicb.2023.1255525

**Published:** 2023-10-02

**Authors:** Enfu Tao, Yuhao Wu, Chenmin Hu, Zhenya Zhu, Diya Ye, Gao Long, Bo Chen, Rui Guo, Xiaoli Shu, Wei Zheng, Ting Zhang, Xinyi Jia, Xiao Du, Marong Fang, Mizu Jiang

**Affiliations:** ^1^Pediatric Endoscopy Center and Gastrointestinal Laboratory, Children’s Hospital, Zhejiang University School of Medicine, National Clinical Research Center for Child Health, National Children's Regional Medical Center, Hangzhou, China; ^2^Department of Neonatology and NICU, Wenling Maternal and Child Health Care Hospital, Wenling, China; ^3^Department of Gastroenterology, Children's Hospital, Zhejiang University School of Medicine, National Clinical Research Center for Child Health, National Children's Regional Medical Center, Hangzhou, China; ^4^Institute of Neuroscience and Gastrointestinal Laboratory, Children’s Hospital, Zhejiang University School of Medicine, Hangzhou, China

**Keywords:** irritable bowel syndrome, early life stress, brain-gut-microbiota axis, maternal separation, visceral hypersensitivity

## Abstract

**Background:**

Irritable bowel syndrome (IBS) is one of the most common functional gastrointestinal disorder. Traditionally, early life stress (ELS) is predisposed to IBS in adult. However, whether ELS induces IBS in early life remains unclear.

**Methods:**

Separated cohort studies were conducted in neonatal male pups of C57BL/6 mice by maternal separation (MS) model. MS and non-separation mice were scheduled to be evaluated for prime IBS-phenotypes, including visceral hypersensitivity, intestinal motility, intestinal permeability, and anxiety-like behavior. Ileal contents and fecal samples were collected and analyzed by 16S rRNA gene sequencing and bacterial community analyses. Subcellular structures of intestinal epithelial, such as epithelial tight junctions and mitochondria, were observed under transmission electron microscopy.

**Results:**

MS induced visceral hypersensitivity and decreased total intestinal transit time from childhood to adulthood. In addition, MS induced intestinal hyperpermeability and anxiety-like behavior from adolescence to adulthood. Besides, MS affected intestinal microbial composition from childhood to adulthood. Moreover, MS disrupted intestinal mitochondrial structure from childhood to adulthood.

**Conclusion:**

The study showed for the first time that MS induced IBS from early life to adulthood in mice. The disrupted intestinal mitochondrial structure and the significant dysbiosis of intestinal microbiota in early life may contribute to the initiation and progress of IBS from early life to adulthood.

## Introduction

Irritable Bowel Syndrome (IBS) is a functional gastrointestinal disorder, newly called “disorders of the gut-brain interaction,” which was characterized by recurrent episodes of abdominal pain/discomfort and bowel habit changes (Botschuijver et al., 2019; Han et al., 2022; Brierley et al., 2023), with high prevalence both in childhood and adulthood worldwide (Sperber et al., 2021). With recurrent symptoms and without particularly effective treatments, IBS significantly affects the quality of life, and mental and physical health of patients (Black and Ford, 2020; Sperber et al., 2021; Li et al., 2023). Despite the major global effort, the mechanism underlying the pathogenesis of IBS remains unknown (Mishima and Ishihara, 2021). Brain-gut axis dysfunction and visceral hypersensitivity are two of the main characteristics of IBS, while intestinal hyperpermeability, abnormal gastrointestinal motility, activation of the intestinal mucosal immune response, low-grade intestinal inflammation, and somatic and psychological disorders may also be involved in the pathophysiological processes (Xiao et al., 2021; Tesfaye et al., 2023). In recent years, more attention has been focused on the role of early life stress (ELS) in the pathogenesis of IBS. A large number of pre-clinical and clinical studies have shown that ELS can result in persistent changes in the central stress response systems, heightened visceral hypersensitivity, enhanced intestinal motility, shifts in gut microbiota composition, elevated anxiety-and depressive-like behaviors, and increase predisposition to developing IBS in adulthood (Riba et al., 2018; Ju et al., 2020; Low et al., 2020; Rincel and Darnaudéry, 2020; Collins et al., 2023; Petitfils et al., 2023; Lee and Jung, 2024). Early life is an important period for the development of the central nervous system (CNS), gut, and gut microbiota (Osadchiy et al., 2019; Ratsika et al., 2021). ELS can disrupt this critical period and may contribute to the etiology of several neurodevelopmental disorders, such as IBS (Osadchiy et al., 2019; Tao et al., 2022b). Accordingly, ELS may impact the brain-gut-microbiota axis before adulthood. However, whether ELS can result in IBS in children and adolescents is not yet understood.

Maternal separation (MS) is a classic animal model of IBS, which effectively mimics ELS (Riba et al., 2018; Wong et al., 2019; Huang S. T. et al., 2021; Tao et al., 2022a). Using MS model, some of the pathogeneses of IBS were widely studied, such as visceral hypersensitivity (Wu et al., 2020; Huang S. T. et al., 2021; Wang et al., 2022; Tao et al., 2022a), intestinal hyperpermeability (Kuti et al., 2020; Torres-Maravilla et al., 2022), intestinal dysmotility (Bülbül and Sinen, 2021), intestinal dysbiosis (Rincel and Darnaudéry, 2020; Park et al., 2021), and anxiety-like and depressive-like behaviors (Zhou et al., 2022; Favoretto et al., 2023). Also, Riba et al. (Riba et al., 2018) systematically studied the influence of MS on the function of the intestine, mimicking IBS’s main features, including intestinal hyperpermeability, visceral hypersensitivity, microbiota dysbiosis, bile acid malabsorption, and low grade inflammation in the intestine. Results suggested that MS is a suitable model for IBS. These studies mainly focused on the effect of MS on adult rodents; however, few studies paid attention to the influence of MS on young rodents. One study reported that MS rats showed significant visceral hypersensitivity from the post-weaning period to adult (Yi et al., 2017). Moreover, our previous study using a novel distention balloon to evaluate visceral hypersensitivity found that MS induced visceral hypersensitivity in post-weaning mice (Tao et al., 2022a). Together, these results suggested that visceral hypersensitivity in the early life, such as post-weaning period, might play a more meaningful pathophysiologic role in the formation of adult IBS. Therefore, to dynamically explore the potential effect of MS on early life to adulthood may provide a new vision of the pathogenesis of IBS, and thus may develop new therapeutic targets for IBS.

Accordingly, we conducted separated cohort studies of mice to investigate the hypothesis that ELS induced prime phenotypes of IBS, such as visceral hypersensitivity, intestinal hyperpermeability, abnormal gastrointestinal motility, intestinal dysbiosis, and anxiety-like behavior, from childhood to adulthood.

## Materials and methods

### Study design

**Figure 1:**
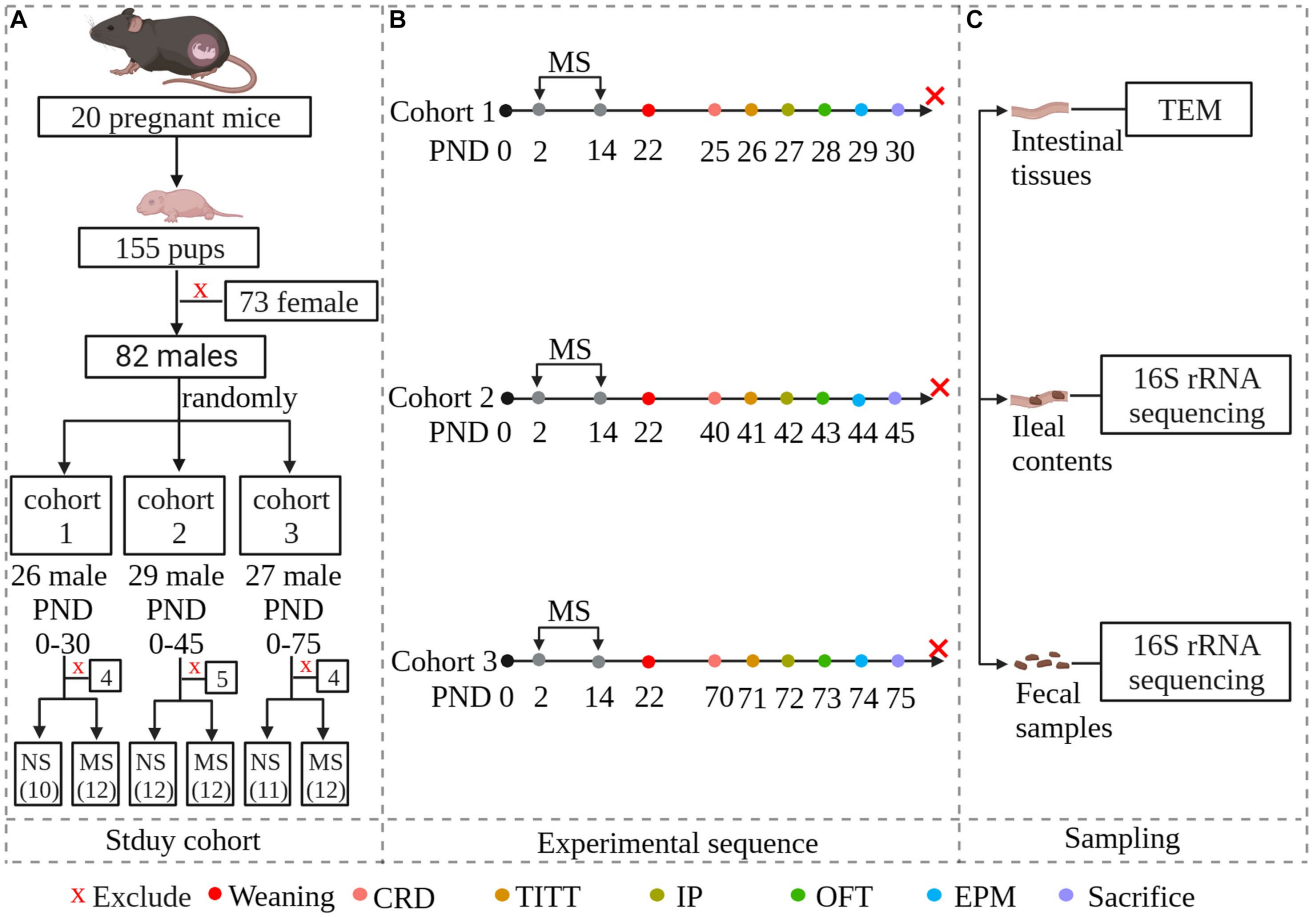
Experimental overview and methodology of the study. The study investigates whether early-life stress induced irritable bowel syndrome during early life stages, including childhood and adolescence, and persists into adulthood. To simulate early-life stress, we established a maternal separation model. Newborn mice were randomly divided into three cohorts: PND 25–30 representing childhood, PND 40–45 representing adolescence, and PND 70–75 representing adulthood. Within each age group, neonatal mice were further randomly divided into maternal separation groups and non-separation groups. Each MS group was experiencing maternal separation at PND 2–14. Both MS and NS groups were weaned at PND 22. Both gut functional and behavioral parameters were evaluated at specific predetermined times. Fecal samples and ileal contents were collected for microbial community analysis by 16S rRNA sequencing. Intestinal tissues were collected and subcellular structures of intestinal epithelial were observed under transmission electron microscopy. **(A)**: Study cohort; **(B)**: Experimental sequence. Animals were scheduled to be evaluated for intestinal parameters and behavior, and then to be sacrificed. **(C)**: Sampling. NS, non-separation; MS, maternal separation; CRD, colorectal distention; TITT, total intestinal transit time; IP, Intestinal permeability; OFT, pen field test; EPM, elevated plus maze; TEM, Transmission Electron Microscopy.

Twenty pregnant C57BL/6 mice of 13 days gestation age were purchased from the Laboratory Animal Center of Zhejiang University. They were individually housed and maintained on a 12-h light–dark cycle (turned on at 9: 00 am and turned off at 9:00 pm) with access to food and water *ad libitum*. To avoid the effects of stress on dams, litters were not disturbed on the first one day after delivery. Female pups were euthanized on postnatal day (PND) 2 by decapitation after being anesthetized with 2% isoflurane. To avoid the effects of estrogen, only male pups (n = 82) were used. Number the entire litters of mice from 1 to 20 and generate random numbers using an Excel spreadsheet. Arrange them in ascending order based on the random numbers. Assign the first 6 random numbers to cohort 1, numbers 7–13 to cohort 2, and numbers 14–20 to cohort 3. After grouping, each cohort was further divided into MS groups and non-separation (NS) groups using the same method. The schematic of the study design was shown in Figure 1. Protocols for animal research were preapproved by the Zhejiang University Ethics Committee for Animal Research (ethics review number: ZJM20230025).

### Maternal separation

MS was implemented as previously described (Riba et al., 2018; Wong et al., 2019; Tao et al., 2022a). For the protocol of MS please refer to the Supplementary data.

### Abdominal withdrawal reflex

Abdominal withdrawal reflex (AWR) score was evaluated by colorectal distension (CRD) on mice at PND 25, 40 and 70 according to the previous study with some modifications (Yu et al., 2012; Zhang Y. et al., 2020; Tao et al., 2022a). For the protocol of AWR please refer to the Supplementary Data.

### Total intestinal transit time

The total intestinal transit time (TITT) was measured by carmine red as previous study used (Schmitt et al., 2017). Briefly, carmine red (1390-65-4, MedChemExpress) was given by gavage to mice fasted for 6 h (10 mg/mL of water, 10 μL/g body weight). The TITT was measured by the time between ingestion of carmine red and first appearance of the dye in feces.

### Intestinal paracellular permeability

Intestinal paracellular permeability was evaluated by the intestinal permeability of fluorescein isothiocyanate-dextran (FITC-D) 4 kDa as previous studies described with some modifications (Toubal et al., 2020; Ye et al., 2021). For the detailed protocol please refer to the Supplementary data.

### Animal behavior experiments

Animal behavior experiments were implemented during the dark phase of the diurnal cycle in the Laboratory Animal Center of Zhejiang University. Mice were placed in the experiments room 0.5 h ahead of experiments for environmental adaptation.

### Open-field test

Open-field test (OFT) was tested as previous study (Chen et al., 2021). For the protocol of OFT please refer to the Supplementary data.

### Elevated plus maze

The protocol of elevated plus maze (EPM) was previous described (Zhang H. et al., 2020). For the protocol of EPM please refer to the Supplementary data.

### Transmission electron microscopy

The protocol of transmission electron microscopy (TEM) was previous reported (Ye et al., 2021). Briefly, ileal fresh tissues about 0.5–1 cm were fixed overnight in 2.5% glutaraldehyde at 4°C. After rinsed three times for 10 min each with PBS, tissues were fixed with 1% osmium tetroxide for 1 h. Then, the specimens were rinsed in distilled water 10 min each for three times, followed by stained with 2% aqueous uranyl acetate for 30 min. The samples were subjected to dehydration in an ethanol gradient series: 50%, 70%, and 90% ethanol, each for 15 min, followed by 100% ethanol for 20 min treatments in shaking table (60 rpm). Then samples were treated with 100% acetone twice for 20 min each. Embedding: pure acetone + embedding solution (1:1) was incubated for 2 h at room temperature, pure acetone + embedding solution (1:3) was incubated for 2 h at room temperature, and then the solution was replaced with pure embedding solution and was embedded at 37°C. After polymerization, 90 nm thick sections were cut using an ultra-microtome (LEICA EM UC7, Leica, United States). Finally, samples were observed by 120kv TEM (Tecnai G2 Spirit 120 kV, Thermo FEI).

### Mitochondrial measurement

Mitochondria morphology within TEM images was analyzed with ImageJ, encompassing mitochondrial length, width, and area, followed previously published approaches (Lam et al., 2021). Mitochondrial cristae were evaluated by a cristae score: 0, no well-defined cristae; 1, more than 50% of the mitochondrial area lacks cristae; 2: more than 25% of the mitochondrial area lacks cristae; 3: many irregular cristae covering over 75% of the area; 4: Many regular cristaer (Eisner et al., 2017).

### Microbiota analysis

Ileal contents and fecal DNA extraction, 16S rRNA gene sequencing, and bacterial community analysis.

For materials and methods, please refer to the Supplementary data.

### Statistical analyses

The distribution of data was analyzed by Shapiro–Wilk normality test. Normally distributed data is represented using the mean ± standard deviation (SD), whereas non-normally distributed data is represented using the median and interquartile range (IQR). Two sets of normally distributed data are analyzed using a Student’s t-test, while non-normally distributed data are analyzed using non-parametric tests. For AWR, two-way repeated-measures ANOVA followed by Bonferronis multiple-comparisons test was used. All data were analyzed by IBM Statistical Package for the Social Sciences (SPSS), version 23 (IBM Corporation). *p* < 0.05 was considered statistically significant.

## Results

### ELS induced visceral hypersensitivity from childhood to adulthood

#### AWR vs. threshold

**Figure 2:**
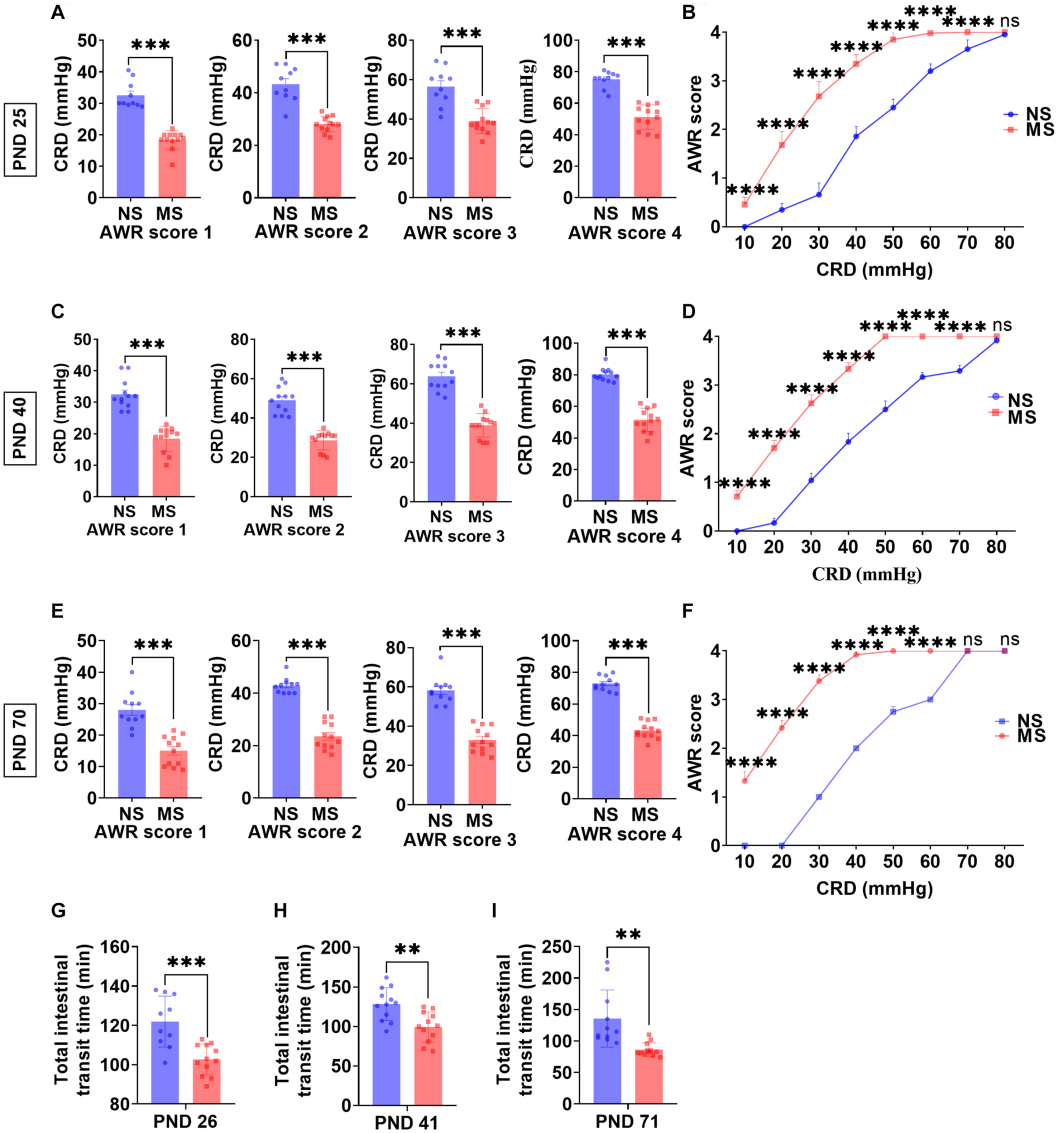
Early life stress induced visceral hypersensitivity and promoted intestinal motility from childhood to adulthood. **(A,B)**: impact of early life stress on visceral sensitivity at PND 25. **(C,D)**: impact of early life stress on visceral sensitivity at PND 40. **(E,F)**: impact of early life stress on visceral sensitivity at PND 70. **(G–I)**: impact of early life stress on intestinal motility at PND 26, 41, and 71, respectively. AWR, abdominal withdrawal reflex; CRD, colorectal distention; NS, non-separation; MS, maternal separation; PND, postnatal day; ns, no significance; **p < 0.01; ***p < 0.001; ****p < 0.0001.

The CRD threshold of AWR score 1, 2, 3, and 4 at PND 25 was significantly lower in MS compared to NS (*p* < 0.0001, respectively) (ELS × pressure) with Bonferronis multiple-comparisons test, interaction: *F* (3, 80) = 2.99, *p* < 0.05; and it had significant main effect of ELS: *F* (1, 80) = 191.2, *p* < 0.0001; also significant main effect of pressure: *F* (3, 80) = 160.2, *p* < 0.0001 (Figure 2A). Besides, the CRD threshold of AWR score 1, 2, 3, and 4 at PND 40 was significantly lower in MS compared to NS (*p* < 0.000, respectively) (ELS × pressure) with Bonferronis multiple-comparisons test, interaction: *F* (3, 88) = 7.27, *p* < 0.001; and it had significant main effect of ELS: *F* (1, 88) = 354.2, *p* < 0.0001; also significant main effect of pressure: *F* (3, 88) = 217.2, *p* < 0.0001 (Figure 2C). In addition, the CRD threshold of AWR score 1, 2, 3, and 4 at PND 70 was significantly lower in MS compared to NS (*p* < 0.0001, respectively) (ELS × pressure) with Bonferronis multiple-comparisons test, interaction: *F* (3, 84) = 10.70, *p* < 0.0001; and it had significant main effect of ELS: *F* (1, 84) = 387.7, *p* < 0.0001; also significant main effect of pressure: *F* (3, 84) = 202.7, *p* < 0.0001 (Figure 2E).

#### AWR vs. pressure

The AWR scores at 10 mm Hg, 20 mmHg, 30 mmHg, 40 mmHg, 50 mmHg, 60 mmHg, and 70 mmHg at PND 25 were significantly higher in MS compared to NS (*p* < 0.0001, respectively) (ELS × pressure) with Bonferronis multiple-comparisons test, interaction: *F* (7, 160) = 80.57, *p* < 0.0001; and it had significant main effect of ELS: *F* (1, 160) = 1,361, *p* < 0.0001; also significant main effect of pressure: *F* (7,160) = 1,364, *p* < 0.0001) (Figure 2B). However, there was no difference of AWR score at 80 mmHg pressure of CRD between MS and NS at PND 25 (*p* > 0.05) (Figure 2B). The AWR scores at 10 mm Hg, 20 mmHg, 30 mmHg, 40 mmHg, 50 mmHg, 60 mmHg, and 70 mmHg at PND 40 were significantly higher in MS compared to NS (*p* < 0.0001, respectively) (ELS × pressure) with Bonferronis multiple-comparisons test, interaction: *F* (7, 176) = 140.9, *p* < 0.0001; and it had significant main effect of ELS: *F* (1, 176) = 4,111, *p* < 0.0001; also significant main effect of pressure: *F* (7,176) = 3,329, *p* < 0.0001) (Figure 2D). However, there was no difference of AWR score at 80 mmHg pressure of CRD between MS and NS at PND 40 (*p* > 0.05) (Figure 2D). The AWR scores at 10 mm Hg, 20 mmHg, 30 mmHg, 40 mmHg, 50 mmHg, and 60 mmHg at PND 70 were significantly higher in MS compared to NS (*p* < 0.0001, respectively) (ELS × pressure) with Bonferronis multiple-comparisons test, interaction: *F* (7, 168) = 872.4, *p* < 0.0001; and it had significant main effect of ELS: *F* (1, 168) = 12,901, *p* < 0.0001; also significant main effect of pressure: *F* (7,168) = 6,200, *p* < 0.0001) (Figure 2F). However, there was no difference of AWR score at 70 and 80 mmHg pressure of CRD between MS and NS at PND 70 (*p* > 0.05) (Figure 2F).

### ELS promoted intestinal motility from childhood to adulthood

The TITT in MS was significantly shorter than NS at PND 26 (102.70 ± 7.83 min *VS* 121.9 ± 12.97 min, *p* < 0.0001, Figure 2G), at PND 41 (99.42 ± 18.88 min *VS* 128.3 ± 20.62 min, *p* < 0.01, Figure 2H) and at PND 71 (86.33 ± 10.76 min *VS* 135.6 ± 45.43 min *p* < 0.01, Figure 2I), respectively.

### ELS increased intestinal paracellular permeability from adolescence to adulthood

**Figure 3:**
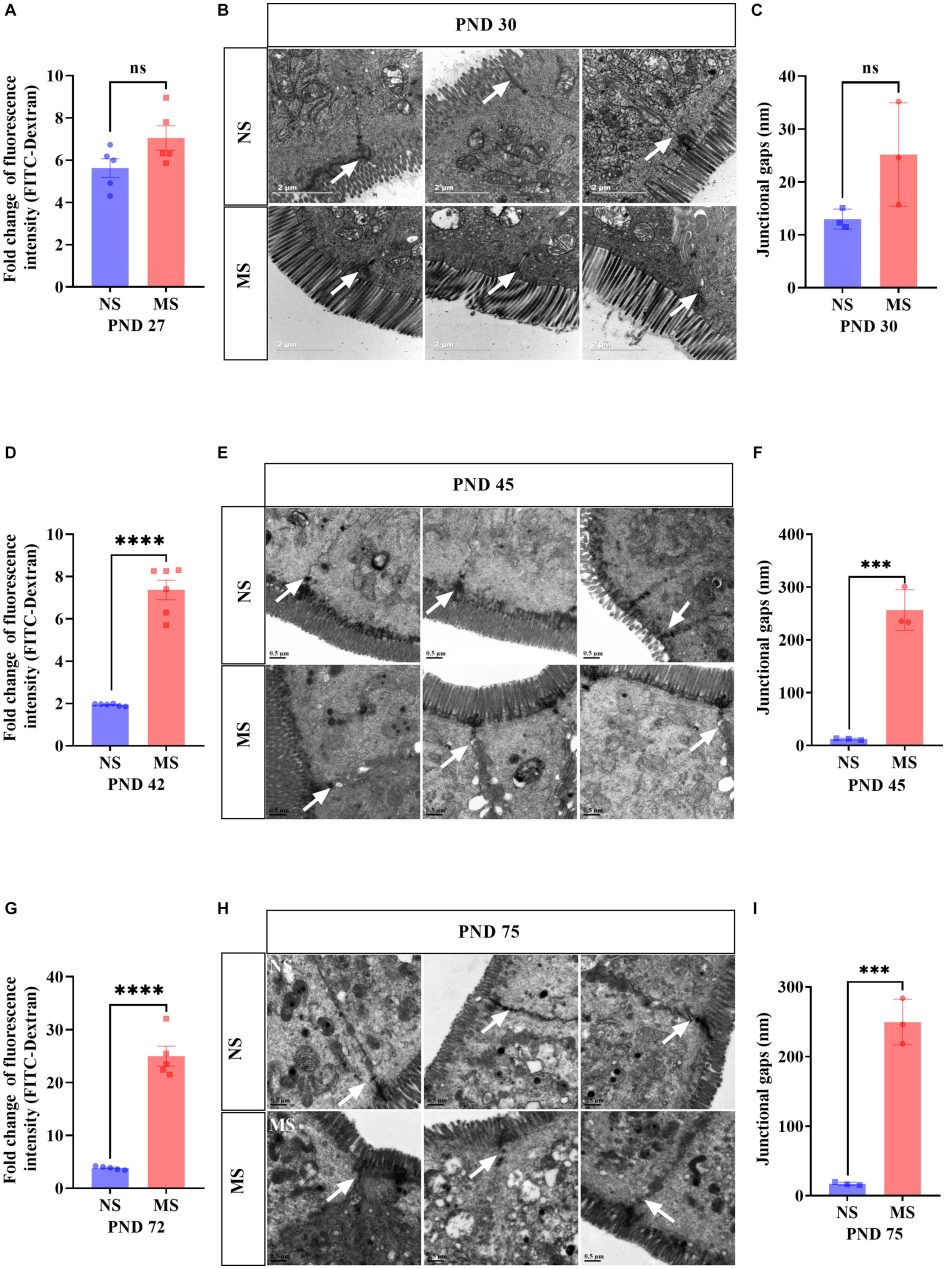
Early life stress induced intestinal hyperpermeability from adolescence to adulthood. **(A,D,G)**: impact of early life stress on intestinal hyperpermeability assessed by FITC-Dextran at PND 27, 42 and 72, respectively. **(B,E,H)**: Intestinal epithelium was observed by transmission electron microscope at PND 30, 45, and 75, respectively. **(C,F,I)**: junctional gaps in the images of transmission electron microscope were measured by ImageJ at PND 30, 45, and 75, respectively. FITC, fluorescein isothiocyanate; NS, non-separation; MS, maternal separation; PND, postnatal day; ns, no significance; ***p < 0.001; ****p < 0.0001. The white arrow indicated tight junctions between intestinal epithelial cells.

Compared to NS, fluorescence intensity of FITC-Dextran in serum in MS was significantly higher at PND 42 (7.37 ± 1.13 *VS* 1.94 ± 0.05, *p* < 0.001, Figure 3D) and PND 72 (24.99 ± 4.24 *VS* 3.86 ± 0.33, *p* < 0.001, Figure 3G), respectively. However, there was no significant difference of fluorescence intensity of FITC-Dextran in serum between MS and NS at PND 27 (*p* > 0.05) (Figure 3A). TEM of intestine tissue revealed that the epithelial tight junctions were loosened and the gap widened in MS compared to NS at PND 45 (256.4 ± 38.47 nm *VS* 12.27 ± 1.67 nm, *p* < 0.001, Figure 3F) and PND 75 (249.5 ± 32.70 nm *VS* 17.30 ± 2.21 nm, *p* < 0.001, Figure 3I). However, there was no significant different junctional gaps between MS and NS at PND 30 (*p* > 0.05) (Figure 3C).

### ELS disrupted intestinal mitochondrial structure

**Figure 4:**
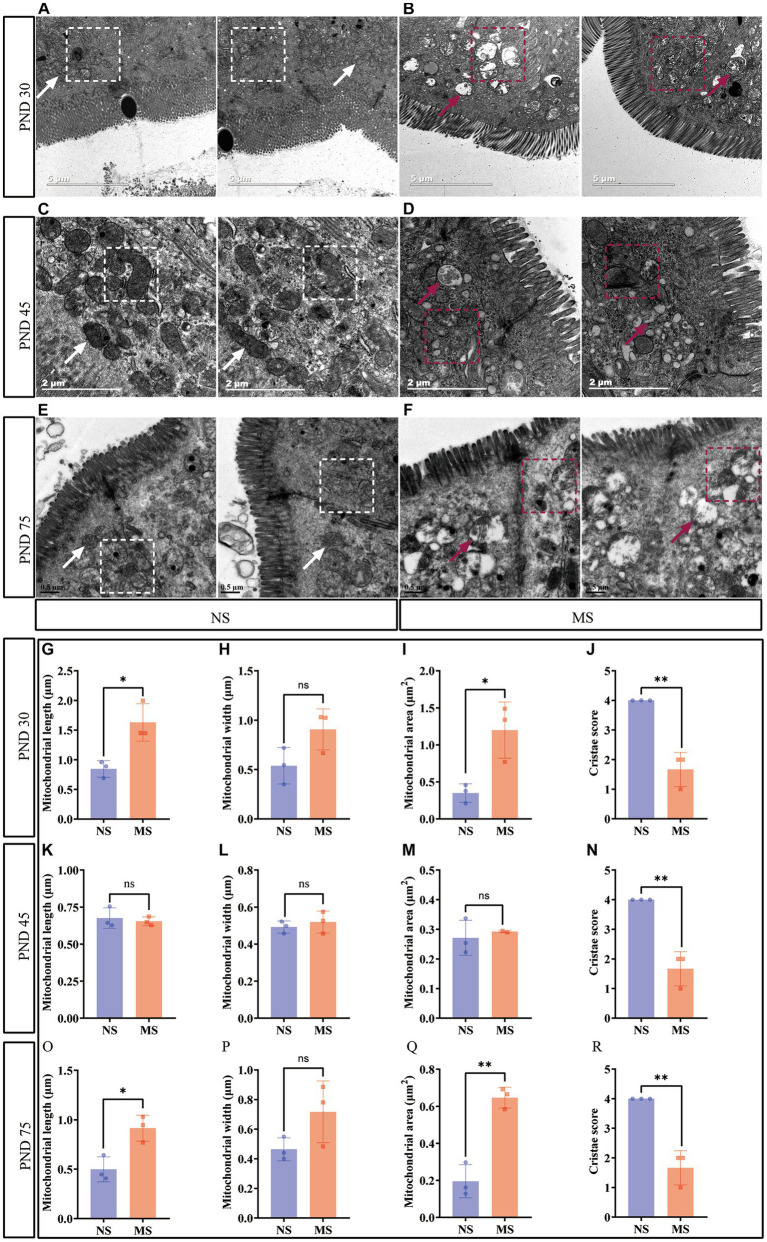
Early life stress disrupted intestinal mitochondrial structure. **(A,B)**: impact of early life stress on intestinal mitochondrial structure at PND 30; **(C,D)**: impact of early life stress on intestinal mitochondrial structure at PND 45; **(E,F)**: impact of early life stress on intestinal mitochondrial structure at PND 75. **(G,H,I)**: mitochondrial measurement, including length, width, and area, at PND 30, respectively. **(K,L,M)**: mitochondrial measurement, including length, width, and area, at PND 45, respectively. **(O,P,Q)**: Mitochondrial measurement, including length, width, and area, at PND 70, respectively. **(J,N,R)**: cristae score at PND 30, 45, and 70, respectively. The white arrow indicated normal morphology of mitochondria and the white dotted box indicated well-organized mitochondrial arrangement in intestinal epithelial cells, with normal mitochondrial cristae morphology. The red arrow and red dotted box indicated irregular mitochondrial size and morphology in intestinal epithelial cells, with the formation of mitochondrial cristae vacuolation. NS, non-separation; MS, maternal separation; PND, postnatal day; ns, no significance; *p < 0.05; **p < 0.01.

Intestinal mitochondrial structure was observed under TEM. The mitochondria were disarranged, irregular in size and shape, and displaying cristae vacuolation in MS (Figures 4B,D,F) (marked with red arrows and red dotted box) compared to NS (Figures 4A,C,E) (marked with white arrows and white dotted box). Mitochondrial morphology in electron microscope images was analyzed using ImageJ. Compared to NS, MS exhibited significantly longer mitochondrial length at PND 30 (1.63 ± 0.32 μm vs. 0.85 ± 0.14 μm, *p* < 0.05) (Figure 4G). Additionally, the mitochondrial area was significantly larger in MS compared to NS at PND 30 (1.20 ± 0.38 μm^2^ *VS* 0.35 ± 0.13 μm^2^, *p* < 0.05, Figure 4I). However, there was no significant difference in mitochondrial width between MS and NS (1.02 (0.67, 1.03) *VS* 0.55 (0.35, 0.72), *p* > 0.05) at PND 30, Figure 4H). There was significant difference in mitochondrial length in MS compared to NS at PND 75 (0.91 ± 0.13 μm vs. 0.50 ± 0.13 μm, *p* < 0.05, Figure 4O). Similarly, the mitochondrial area was significantly larger in MS than in NS at PND 75 (0.65 ± 0.056 μm^2^ *VS* 0.20 ± 0.09 μm^2^, *p* < 0.01, Figure 4Q). However, there was no significant difference in mitochondrial width between the two groups at PND 75 (0.72 ± 0.21 μm *VS* 0.46 ± 0.08 μm, *p* < 0.05, Figure 4P). In addition, there were no significant differences in terms of mitochondrial length, width and area between MS and NS at PND 40 (*p* > 0.05, Figures 4K,L,M, respectively). Notably, the cristae scores were significantly lower in MS group compared to NS group at PND 30 (1.67 ± 0.58 *VS* 4.0 ± 0.00, *p* < 0.01, Figure 4J), 45 (1.67 ± 0.58 *VS* 4.0 ± 0.00, *p* < 0.01, Figure 4N), and 75 (1.67 ± 0.58 *VS* 4.0 ± 0.00, *p* < 0.01, Figure 4R).

### ELS induced anxiety-like behavior from adolescence to adulthood

**Figure 5:**
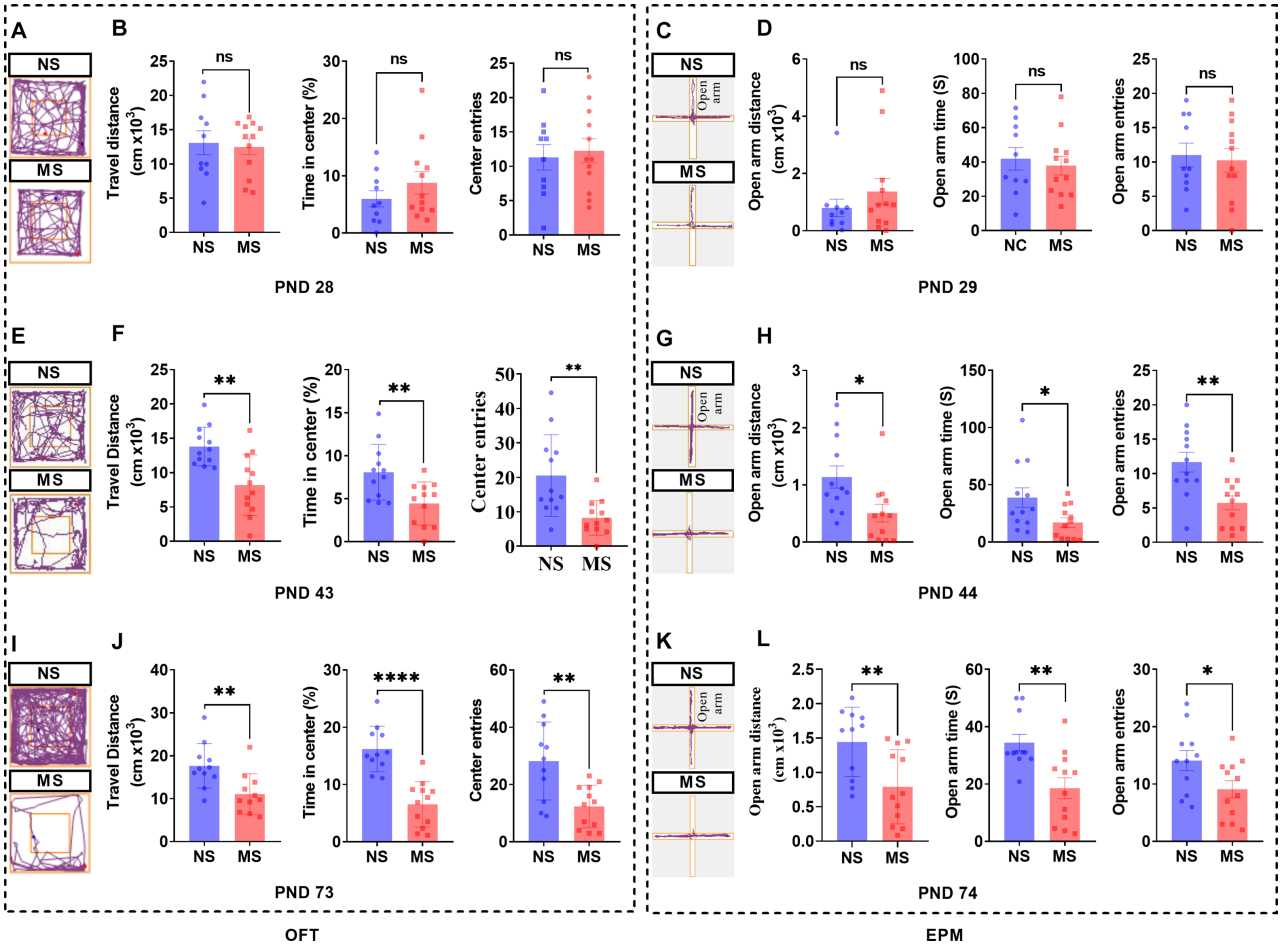
Early life stress induced anxiety-like behavior from adolescence to adulthood in mice. **(A,E,I)**: track diagram of OFT; **(C,G,K)**: track diagram of EPM; **(B,F,J)**: performance in OFT at PND 28, 43, and 73, respectively; **(D,H,L)**: performance in EPM at PND 28, 43, and 73, respectively. ns, no significance; NS, non-separation; MS, maternal separation; PND, postnatal day; OFT, open field test; EPM, elevated plus maze. *p < 0.05; **p < 0.01; ****p < 0.0001.

There were no differences in the performance of OFT (Figures 5A,B) at PND 28 and of EPM (Figures 5C,D) at PND 29 between MS and NS groups (*p* > 0.05, respectively). However, there were significant differences in the performance of OFT, including shorter travel distance, lesser percent time in center, and lesser center entries in MS compared to NS groups at PND 43 (*p* < 0.01, respectively) (Figures 5E,F). Also, there were significant differences in the performance of EPM, including shorter open arm distance (*p* < 0.05), decreased open arm time (*p* < 0.05), and lesser open arm entries (*p <* 0.01) respectively, in MS compared to NS groups at PND 44 (Figures 5G,H). Likewise, there were significant differences in the performance of OFT, including shorter travel distance (*p* < 0.01), lesser percent time in center (*p* < 0.01) and lesser center entries (*p* < 0.001), respectively, in MS compared to NS groups at PND 73 (Figures 5I,J). Also, there were significant differences in the performance of EPM, including shorter open arm distance, decreased open arm time, and lesser open arm entries in MS compared to NS groups at PND 74 (*p* < 0.01, respectively) (Figures 5K,L).

### Impact of ELS on microbial composition of ileal contents at genus level

**Figure 6:**
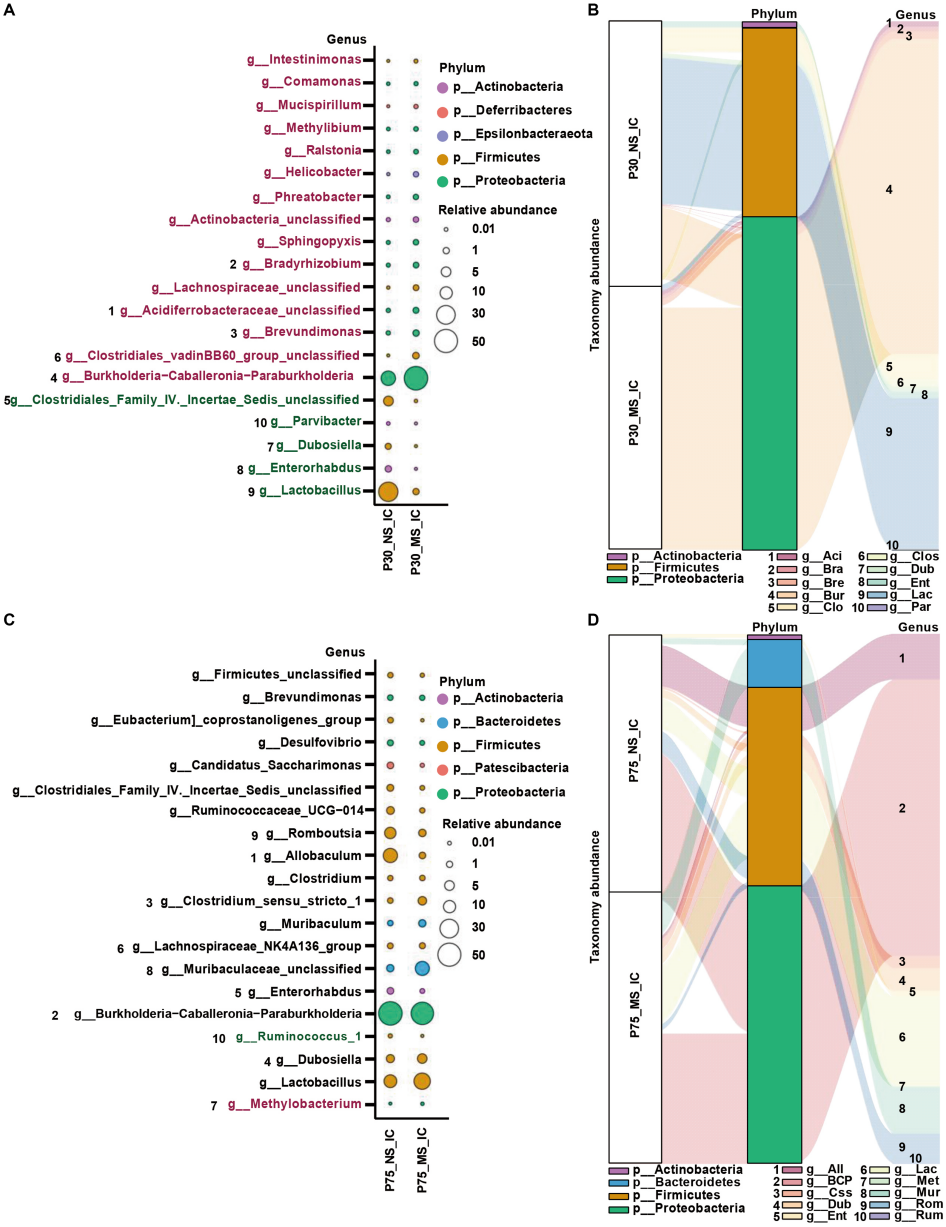
Impact of early life stress on microbial community of ileal contents at genus level from PND 30 to PND 75. **(A,C)** Bubble plot showed the significant difference of microbial abundance of ileal contents between MS and NS at Genus level at PND 30 and PND 75, respectively. **(B,D)** Sankey diagram showed significant differences of taxonomy abundance of microbial community of ileal contents between MS and NS at PND 30 and PND 75, respectively. The upregulated microbial abundance was marked with red, while the downregulated microbial abundance was marked with green. Arabic numeral (1–10) represented different Genus. Genus names that were abbreviated by their first three or four letters in Sankey diagram could be found in Bubble plot.

Concerning the genus level, *Burkholderia-Caballeronia-Paraburkholderia*, *Brevundimonas*, *Bradyrhizobium*, *Clostridiales_vadinBB60_group_unclassified*, *Acidiferrobacteraceae_unclassified*, *Lachnospiraceae_unclassified*, *Sphingopyxis*, *Actinobacteria_unclassified*, *Phreatobacter*, *Helicobacte* were significantly more enriched in the MS group compared to NS group (Figures 6A,B). On the contrary, the abundance of *Lactobacillus*, *Parvibacter*, *Enterorhabdus*, *Dubosiella,* and *Clostridiales_Family_IV._Incertae_Sedis_unclassified* was significantly decreased in the MS group compared to NS group (Figures 6A,B). Furthermore, the abundance of *Ruminococcus_1* was significantly decreased and the abundance of *Methylobacterium* was significantly enriched in the MS group compared to the NS group at the genus level (Figures 6C,D).

### Impact of ELS on microbial composition of fecal samples at genus level

**Figure 7:**
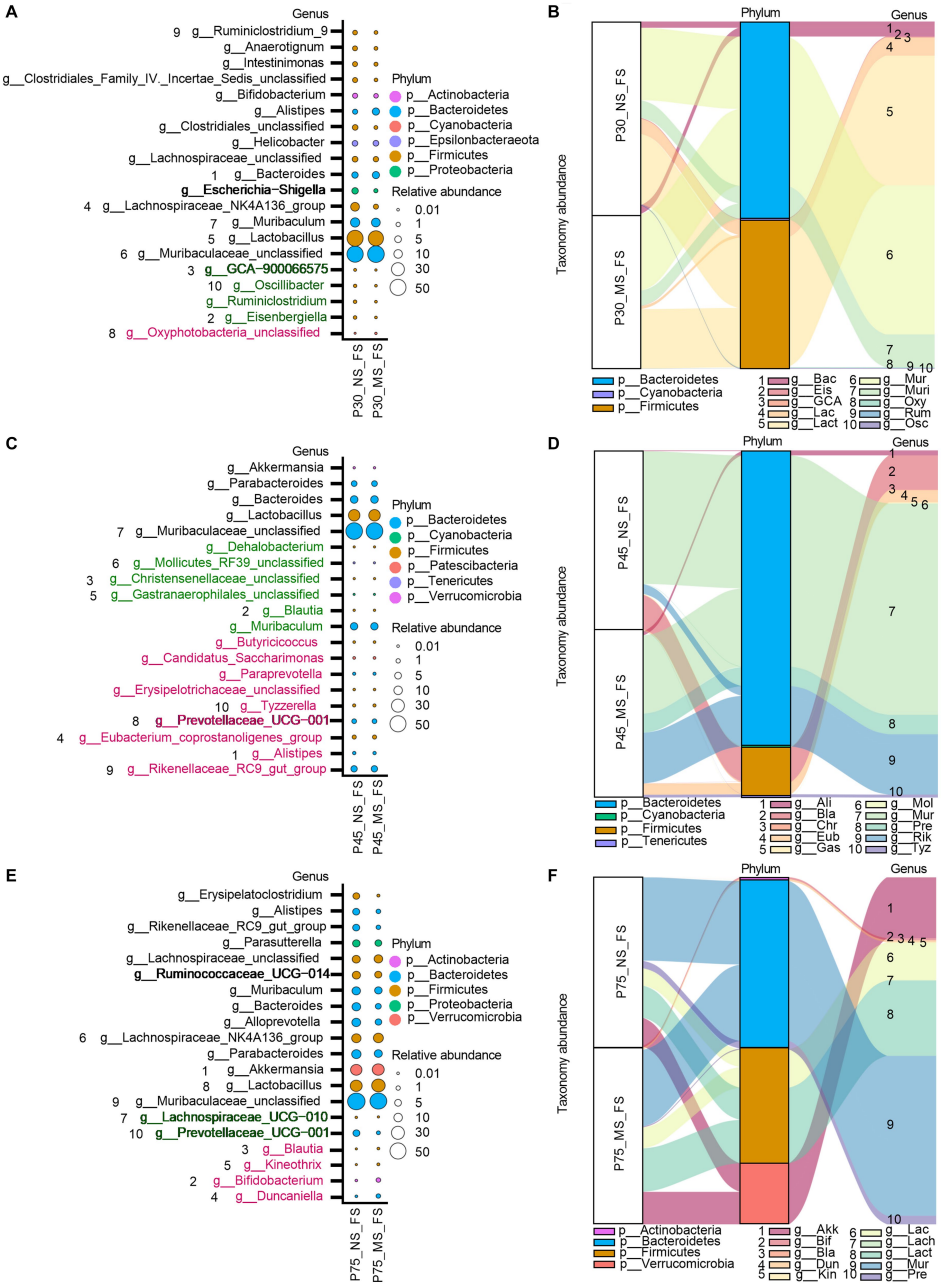
Impact of early life stress on microbial community of fecal samples at genus level from PND 30 to PND 75. **(A,C,E)**: bubble plot showed the significant difference of microbial abundance of fecal samples at genus level at PND 30, 45, and 75, respectively. **(B,D,F)**: Sankey diagram showed significant differences of taxonomy abundance of microbial community of fecal samples between MS and NS at PND 30, 45, and PND 75. The upregulated microbial abundance was marked with red, while the downregulated microbial abundance was marked with green. Arabic numeral (1–10) represented different Genus. Genus names that were abbreviated by their first three or four letters in Sankey diagram could be found in Bubble plot.

At genus level, ELS remarkably affect microbial composition of fecal samples from PND 30, PND 45, to PND 75. AS shown in Figure 7, We observed that the MS group had a higher relative proportion of *Oxyphotobacteria_unclassified* at PND 30 (Figures 7A,B), a higher relative enrichment of *Prevotellaceae_UCG-001*, *Tyzzerella*, *Rikenellaceae_RC9_gut_group*, *Paraprevotella*, *Erysipelotrichaceae_unclassified*, *Eubacterium_ventriosum_group*, *Candidatus_Saccharimonas*, *Butyricicoccus*, *Alistipes* at PND 45 (Figures 7C,D), and a higher relative abundance of *Kineothrix*, *Blautia*, *Bifidobacterium*, *Duncaniella* at PND 75 (Figures 7E,F) than that of NS group. However, we found that compared to NS group, MS group had a lower relative composition of *Kineothrix*, *Eisenbergiella*, *GCA-900066575*, *Ruminiclostridium*, *Oscillibacter*, *A2*, and *Lachnospiraceae_UCG-006* at PND 30 (Figures 7A,B), a lower relative component of *Gastranaerophilales_unclassified*, *Mollicutes_RF39_unclassified*, *Muribaculum Dehalobacterium*, *Christensenellaceae_unclassified*, *Blautia*, at PND 45 (Figures 7C,D) and a lower relative proportion of *Prevotellaceae_UCG-001* and *Lachnospiraceae_UCG-010* at PND 75 (Figures 7E,F).

## Discussion

This study investigated the impact of ELS on the main phenotypes of IBS, such as visceral hypersensitivity, hyperpermeability, intestinal dysmotility, intestinal dysbiosis, and anxiety-like behavior from childhood to adulthood. Most importantly, this study showed for the first time, that ELS induced IBS from early life to adult in mice.

Numerous previous basic studies reported that neonatal pups experience of ELS was predisposed to IBS in adulthood (Rincel and Darnaudéry, 2020; Tao et al., 2022b). However, seldom study focused on the impact of ELS on the early life. A large number of clinical evidence indicated that early life adverse event increased the risk for IBS in adult (Burke et al., 2017; Ju et al., 2020; Low et al., 2020). Chang et al. investigated different types of early adverse life events before age 18 years and their association with IBS and demonstrated that IBS patients had a higher prevalence of general trauma, physical punishment, emotional abuse, and sexual events, compared with controls (Bradford et al., 2012). Furthermore, ELS was even correlated with symptom severity of IBS (Park et al., 2016). These clinical studies indicated that ELS play a crucial role in the initiation and progression of IBS. One cannot help but wonder when did ELS cause IBS? Gut microbiota and brain development begin during the prenatal period and continue throughout adulthood, particularly the first 3 years of life representing a critical developmental period. Disruptions in development can influence interaction between these two systems and may contribute to the pathogenesis of neurodevelopmental disorders such as IBS (Borre et al., 2014; Ratsika et al., 2021; Chen et al., 2022). Therefore, there is reason to believe that ELS will disrupt the vital developmental window and may present IBS-like alterations in early life. Indeed, one study supported the idea that ELS caused visceral hypersensitivity from the post-weaning period to adult in rats (Yi et al., 2017). Furthermore, our previous study reported that ELS induced visceral hypersensitivity in post-weaning mice (Tao et al., 2022a). The results indicated that ELS caused IBS-like phenotype in early life. However, beyond these two studies, no other documented study focused on the influence of ELS on IBS-like phenotypes in early life. Accordingly, the profound significance of our study was that it was the first study to comprehensively investigate the influence of ELS on the prime IBS-like phenotypes in early life. Also, our study provided convincing evidence that ELS induced IBS from early life to adult.

In this study, a significant observation was the impact of ELS on the structural integrity of intestinal mitochondria. The mitochondria exhibited evident disruptions in their usual architecture, which included disorganized arrangement, irregular variations in size and shape, and notably, the presence of cristae vacuolation (Figure 4). This significant result indicated that intestinal mitochondrial dysfunction may play an important role in the prosses of ELS induced IBS. Normal mitochondrial function is essential for intestinal epithelial cell homeostasis. Mitochondrial function emerges as a key player in cell fate decisions and in coordinating cellular metabolism, immunity, stress responses and apoptosis (Rath et al., 2018; Guerbette et al., 2022). Preclinical evidence demonstrated that alterations in mitochondrial function and structure are linked to both early stress and systemic biological dysfunction. In addition, early clinical studies supported that increased mitochondrial DNA content and altered cellular energy demands may be present in individuals with a history of ELS (Zitkovsky et al., 2021). Evidence from rodent models suggested that mitochondria exhibited structural and functional changes, such as decreased respiratory enzymatic activity or mitochondrial membrane potential, was associated with long-term or excessive exposure to stress, resulting in an impaired capacity for energy production (Picard and McEwen, 2018; Zitkovsky et al., 2021). Furthermore, chronic psychosocial stress induced epithelial hyperpermeability and visceral hypersensitivity and disturbing mitochondrial activity throughout the intestine (Vicario et al., 2012). Therefore, the ELS induced visceral hypersensitivity (Figure 2), and intestinal hyperpermeability (Figure 3) in early life at the present study may be associated with the dysfunction of mitochondria in early life in intestinal epithelium (Figure 4). Further studies specifically investigating these interactions are warranted. Notably, fluorescence intensity of FITC-D in MS was higher than NS at PND 27, but the difference had no statistical significance (Figure 3A). Subsequent TEM revealed that epithelial tight junctions were loosened and the gap widened in MS compared to NS at PND 30, but the differences were not statistically significant (Figures 3B,C). These results implied that intestinal paracellular permeability was mild increased caused by ELS, but not increased enough to allow biological macromolecules such as FITC-D, entrance. These results also indicated that although the dysfunction of mitochondria was induced by ELS in childhood, the impairment of intestinal barrier function is not severe. On the other hand, work in animal models supported a causal association between mitochondrial dysfunction and changes representative of psychopathology, such as anxiety or depressive-like behaviors (Hollis et al., 2015; Kasahara et al., 2016). MS induced behavioral abnormalities in rats were associated with mitochondrial dysfunction in brain (Khorjahani et al., 2020). Recently, clinical investigations have revealed that significant interactions of mitochondrial respiratory and the inflammatory in the development of anxiety and depression (Liu et al., 2023). We found that MS mice presented anxiety-like behavior at adolescence and adulthood, but not at childhood (Figure 5). These results suggest that behavioral abnormalities induced by ELS may be relatively mild during the early stages of life. Furthermore, the association between abnormal mitochondrial function in the intestines and behavioral anomalies implies that the impact of ELS on intestinal mitochondrial function during early life, such as childhood, might also be subtle. This would also imply that the impact of ELS on intestinal function in early life might be reversed by effective treatment. Therefore, further investigations are much warranted to determine whether early treatment, such as restoring mitochondrial function, can reverse ELS induced IBS-like alterations. However, confirming these hypotheses would require further research.

Intestinal dysbiosis plays an important role the pathogenesis of IBS (Canakis et al., 2020; Petitfils et al., 2023; Zhou et al., 2023). Early life is the critical window for the development of gut microbiota, gut and brain (Laursen et al., 2021; Ratsika et al., 2021). Therefore, disturbance of this process, such as caused by ELS, may have a high predisposition to the development of IBS in adulthood (Wu et al., 2020). We found that MS affected the composition of both ileal and fecal microbiota (Figures 6, 7), suggesting MS affected intestinal microbiota community from early life to adulthood. Importantly, MS significantly reduced the ileal genus abundance of beneficial bacteria, such as *Lactobacillus* (Zhang Q. et al., 2021)*, Parvibacter* (Liu et al., 2021), *Enterorhabdus* (Pagliai et al., 2020), *Dubosiella* (Yuan et al., 2021), and remarkably increased the genus increment of harmful bacteria, such as *Burkholderia-Caballeronia-Paraburkholderia,* at PND 30 (Figures 6A,B). *Lactobacillus and Dubosiella* were short-chain-fatty acids (SCFAs) producing bacteria. The reduction in abundance of *Lactobacillus* and *Dubosiella* may indicate the decreased synthesis of SCFAs level in ileum. SCFAs were important fuels for intestinal epithelial cells (IEC) and regulate IEC functions through different mechanisms to modulate their proliferation, differentiation as well as functions of subpopulations such as enteroendocrine cells, to impact gut motility and to strengthen the gut barrier functions as well as host metabolism (Yao et al., 2020; Martin-Gallausiaux et al., 2021). Stanton et al. found that MS rats had significantly lower ratios of SCFAs producers (Egerton et al., 2020). Additionally, animal exposure to prolonged restraint significantly reduced SCFAs, and *Lactobacillus* in the gut was significantly reduced (Maltz et al., 2018). Furthermore, low SCFAs were reported to be associated with visceral hypersensitivity in rats (Zhang J. D. et al., 2021). Therefore, the intestinal dysbiosis in early life observed in our MS mice might play an important role in the initiation of IBS. Thus, further studies were warranted. On the other hand, the relative abundance of genus *Methylobacterium* in ileum displayed significantly higher in MS than NS at PND 75 (Figures 6C,D). *Methylobacterium* was shown to be related to constipation (Cao et al., 2017). Matsumoto, et al. indicated that genera of *Methylobacterium* was significantly higher in the constipation-predominant IBS compared to diarrhea-predominant IBS (Matsumoto et al., 2021). The pathogenic mechanism by which *Methylobacterium* leads to IBS may be related to its ability to trigger intestinal immune and inflammatory responses (Sun et al., 2021). The role of *Methylobacterium* in the implication of MS induced IBS requires further exploration. In addition to ileum dysbiosis caused by MS, the microbial community of fecal samples was also disturbed from the early life to adulthood (Figure 7). For example, the abundance of *oxyphotobacteria_unclassified* was upregulated in MS compared to NS. Moreover, the abundance of *Lachnospiraceae_UCG-006, Oscillibacter, Ruminiclostridium, GCA-900066575* was downregulated in MS compared to CT at PND 30. Besides, various harmful bacteria were enrichment in fecal samples at PND 45, such as *Candidatus_Saccharimonas* (Cruz et al., 2020)*, Prevotellaceae_UCG-001*(Ibrahim et al., 2019), *Rikenellaceae RC9 gut group* (Emami et al., 2021), *Tyzzerella* (Huang J. et al., 2021)*, Paraprevotella* (Yoon et al., 2021), *Eubacterium_coprostanoligenes_group* (Wei et al., 2021), and the acetic acid production bacteria, such as *Alistipes* (Xu et al., 2021). It reported that *Alistipes* was enriched in post inflammatory irritable bowel syndrome (PI-IBS) (Song et al., 2020). In pediatric patients with IBS, a greater frequency of pain correlated with an increased abundance of the genus *Alistipes* (Saulnier et al., 2011). The results demonstrated that *Alistipes* may play a role in MS induced IBS in early life. Moreover, the abundance of genus *Erysipelotrichaceae_unclassified* was also higher in MS than NS, which was correlation with obesity (Oh et al., 2021). By contrast, some beneficial bacteria, including *Gastranaerophilales_unclassified* (Wu et al., 2021), *Dehalobacterium* (Gu et al., 2020), *Christensenellaceae* (Brandsma et al., 2019), *Blautia* (Wang et al., 2019), *Muribaculum* (McNamara et al., 2021), were reduced in MS compared to NS. *Gastranaerophilales* have been identified as the primary producers of indole, which can subsequently be converted into indolepropionic acid. Indolepropionic acid is recognized for its anti-inflammatory properties in both the gastrointestinal tract and the peripheral system (Rosario et al., 2021). A reduction in *Dehalobacterium* has been linked to the development of inflammation (Chen et al., 2023). The aboundance of *Christensenellaceae* has shown a positive association with microbial metabolites related to intestinal barrier function (Zhao et al., 2022). *The role of Blautia, or its products, in modulating intestinal epithelium health has been highlighted* (Rashidi et al., 2022). Moreover, recent research indicated that *Muribaculum* exhibited a negative correlation with plasma TNF-α and colon inflammatory gene expression (TNF-α), while showing a positive correlation with colon tight junction genes (OCLN and CLDN1) (Yang et al., 2023). Likewise, MS significantly affect the composition of microbiota in fecal samples at PND 75. At the genus level, the beneficial bacteria, including *Prevotellaceae_UCG-001* (Hu et al., 2020) and *Lachnospiraceae_UCG-010* (Han et al., 2021), were downregulated, while the bacteria, such as *Kineothrix* (Xie et al., 2021), *Blautia* (Wang et al., 2019), *Bifidobacterium* (Liu et al., 2021), *Duncaniella* (Chang et al., 2021) were upregulated. *Prevotellaceae_UCG-001* was widely known as a probiotic for superior SCFAs production capacity. A decrease in its abundance has been linked to depression (Zhang Z.-W. et al., 2022) and intestinal inflammation (Wu et al., 2023). *On the other hand, the abundance of Lachnospiraceae_UCG-010 was found to be significantly reduced in the fecal samples of patients with IBS, while it was observed to be increased in healthy individuals* (Zhuang et al., 2018). Study revealed that higher levels of *Lachnospiraceae_UCG-010* were associated with improved intestinal barrier function and a reduction in intestinal lesion scores (Kan et al., 2021). Taken together, MS leads to dysbiosis in fecal samples from early life to adulthood in mice. These findings suggest that early-life exposure to MS disrupts the balance of intestinal microbiota. The observed dysbiosis in the gut microbiota during early life could potentially play a role in the onset and development of MS-induced IBS. Further studies are required to fully elucidate the mechanisms underlying this relationship.

**Figure 8:**
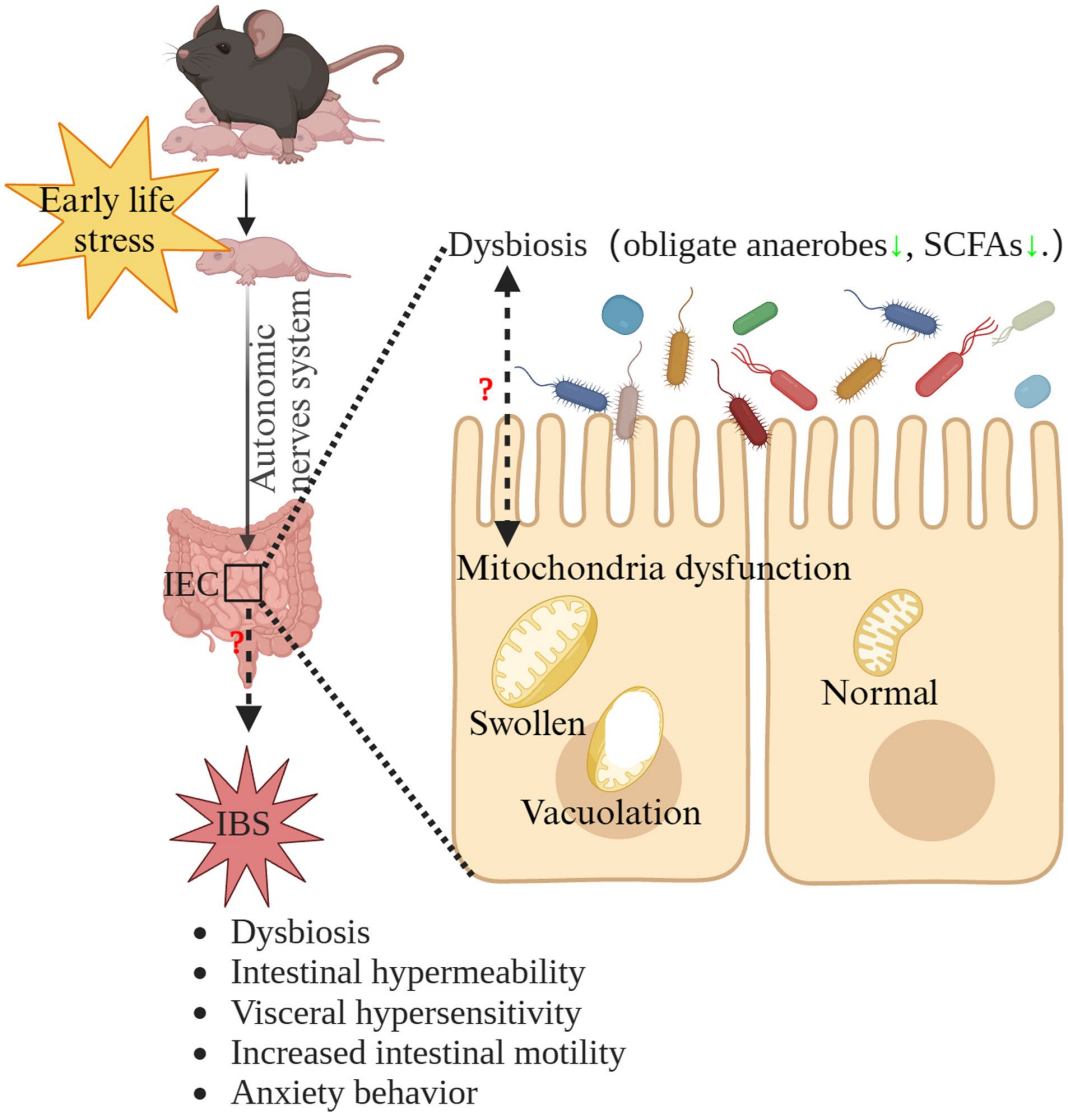
The hypothetical mechanism of the potential role of microbiota-mitochondria crosstalk dysfunction in the pathogenesis of ELS-induced IBS. ELS, early life stress; IBS, irritable bowel syndrome; IEC, intestinal epithelial cells; SCFAs, short-chain-fatty acids.

IBS is a highly prevalent, chronic disorder that significantly reduces patients’ quality of life (Lacy et al., 2021). At present, the pathogenesis of IBS remains elusive and the treatments for IBS are unsatisfactory (van Orten-Luiten et al., 2022; Gao et al., 2023). Therefore, other line of thought may be needed, namely to investigate the initiation and process of IBS. Our results suggested that MS induced IBS in early life. Therefore, when investigating the pathogenesis of IBS, it is necessary to extend the research timeline to the earlier stages of life, which also represents a crucial developmental window for the brain-gut-microbiota axis (Leyrolle et al., 2021). While we did not delve into the potential mechanisms underlying the induction of IBS by ELS, our findings suggest that mitochondrial dysfunction in the intestinal epithelium and dysbiosis of the gut microbiota might play crucial roles in ELS-induced IBS. The interplay between mitochondria and gut microbiota holds a vital significance in maintaining intestinal physiological balance. Under normal circumstances, intestinal epithelial cells plays a vital role in maintaining the hypoxic environment in the intestinal lumen, facilitating the prevalence of obligate anaerobic microbiota, which is dependent of mitochondrial oxidative phosphorylation (Litvak et al., 2018). On the other hand, these microbes contribute essential metabolites like SCFAs to supply nutrients for gut epithelial cells (Takihara and Okuda, 2023). In addition, SCFAs are potentially effective modulators for mitochondria function (Zhang Y. et al., 2022). Microbial communications with intestinal epithelial mitochondria might modify mitochondrial structural characteristics and metabolic capabilities. This abnormal interaction could trigger inflammasome activation, potentially disrupting epithelial hypoxia and affecting the structure of the gut microbiota (Zhang Y. et al., 2022). Interestingly, we found disrupted intestinal mitochondrial structure and the significant dysbiosis of intestinal microbiota in early life. Therefore, microbiota-mitochondria crosstalk dysfunction may be involved in the pathogenesis and initiation of IBS induced by ELS (Zhang Y. et al., 2022). Further research is needed to validate this hypothesis. The hypothetical mechanism of the potential role of microbiota-mitochondria crosstalk dysfunction in the pathogenesis of ELS-induced IBS is summarized in Figure 8.

In conclusion, this study shows for the first time that ELS induces IBS from early life to adulthood in mice. The disrupted intestinal mitochondrial structure and the significant dysbiosis of intestinal microbiota in early life may contribute to the initiation and progress of IBS from early life to adulthood. A noteworthy implication of our study is that it paves the way for new insights into pathogenetic investigation of IBS and contributes to develop novel therapeutic targets for IBS in future investigations.

## Data availability statement

The datasets presented in this study can be found in online repositories. The names of the repository/repositories and accession number(s) can be found at: https://www.ncbi.nlm.nih.gov/, PRJNA804655.

## Ethics statement

The animal study was approved by Zhejiang University Ethics Committee for Animal Research. The study was conducted in accordance with the local legislation and institutional requirements.

## Author contributions

ET: Conceptualization, Methodology Writing – original draft, Writing – review & editing. YW: Methodology, Resources, Writing – review & editing. CH: Methodology, Resources, Writing – review & editing. ZZ: Methodology, Resources, Writing – review & editing. DY: Data curation, Methodology, Writing – review & editing. GL: Data curation, Formal analysis, Writing – review & editing. BC: Writing – review & editing. RG: Writing – review & editing. XS: Data curation, Formal analysis, Writing – review & editing. WZ: Data curation, Formal analysis, Writing – review & editing. TZ: Formal analysis, Visualization, Writing – review & editing. XJ: Data curation, Formal analysis, Writing – review & editing. XD: Data curation, Formal analysis, Writing – review & editing. MF: Formal analysis, Methodology, Writing – review & editing. MJ: Funding acquisition, Supervision, Writing – original draft, Writing – review & editing.
