# Supplementary material for: Early life stress induces irritable bowel syndrome from childhood to adulthood in mice
Source: Front Microbiol. 2023 Oct 2;14:1255525. doi: 10.3389/fmicb.2023.1255525 (PMC10577190; doi:10.3389/fmicb.2023.1255525)
Supplement: Supplementary file 1 [file Data_Sheet_1.docx]

Supplementary data

Materials and Methods

Maternal separation

Briefly, on PND 2-14, MS pups were separated from dams daily from 9:00 am to 12:00 am. They were individually placed into a plastic box (size 180 × 130 × 55 mm) in a separated room. Wood chip bedding was used and maintained ambient temperature of 26 ± 2 ℃. The bedding was changed weekly. After separation, MS pups were back to dams and left undisturbed, whereas non-separation (NS) pups were nursed without disturbance except the change of bedding and the addition of food and water. On PND 15-21, both MS and NS pups were stayed with dams.

Intestinal permeability

Briefly, 6 h water-fasted mice were gavaged with FITC -D (MW 4000; 60842-46-8, MedChemExpress). After 1h, 200 μl of blood were collected from each mouse from the retro-orbital vein (1). Before sampling, mice were anesthetized by 2% isoflurane for 3 minutes. The collected blood was centrifuged at 4000 rpm for 10 min at 4 °C. Plasmic FITC-D fluorescence intensity was analyzed with a fluorescence spectrophotometer (SynergyMx M5, Molecular Devices, USA) at excitation and emission wavelengths of 485 nm and 535 nm, respectively.

Abdominal withdrawal reflex

Briefly, mice were allowed free access to food and water before the experiment. Anuses were lubricated with Vaseline ahead of experiment. The distention balloon (3mm diameter or 5mm, 1cm long) was introduced into the rectum at 1.0 cm from the anus and fixed at the base of the tail under 1.5% isoflurane (RWD Life Science Co.) anaesthesia. Then, mice were placed in a platform and allowed to recover 30 min fully from the anaesthesia and adaption of the balloon. Mice were evaluated with different size balloons according to age, 3mm for childhood and 5mm for adolescence and adulthood.

The AWR scores were graded on a scale of 0-4: 0, no behavioral response to CRD; 1, brief head movement followed by immobility; 2, contraction of abdominal muscles; 3, lifting of abdomen; 4, body arching and lifting of pelvic structures. For measuring the pressure threshold of AWR score 1, 2, 3, and 4, the colorectal balloon was progressively inflated, from 0 mmHg of pressure to the max pressure, and different degrees of pain behaviors (0-4) displayed and the real-time pressure was recorded. When recording, ceased inflation of balloon but keep the pressure. In this manner, the pressure threshold of AWR score 1, 2, 3, and 4 was determined. After 5 minutes of rest, the repeated measurement was performed. Then the mice had 5 minutes of rest for measuring the AWR. For measuring the AWR at different pressures of the balloon, the balloon was rapidly inflated to constant pressure (10, 20, 30, 40, 50, 60, 70 and 80 mmHg). Each pressure was maintained for 5 seconds, and afterwards, the balloon was rapidly and completely deflated. After 20 seconds of rest, the next pressure was performed. In this way, AWR at the pressures of 10-80 mmHg was determined in sequence. Likewise, after 5 minutes of rest, the repeated measurement was performed. Pressures always presented from lowest to highest. All measurements were carried out by 2 blinded observers. The average value of the measurements (the pressure threshold of AWR score 1, 2, 3, and 4 and AWR score at the pressure of 10, 20, 30, 40, 50, 60, 70 and 80 mmHg) was recorded respectively.

Open-field test

Two mice were respectively put into a separate square plastic 45 × 45 × 45 cm^3^ arena with two overhead video tracking system for simultaneously tracing two independent mice movement and recording. The light intensity used in the OFT was 15 ~ 20 Lux. Mice were monitored with computerized tracking software. The duration of the procedure is 5 minutes. Distance moved, time spent moving, time spent in center, and crosses into center were recorded. Between each test, arena was thoroughly cleaned with 70% alcohol, and competed mice were placed in a new cage rather than back with their cage mates. Recorded data were analyzed with ANY-maze software.

Elevated plus maze

The EPM was composed of four elevated (30 cm) arms (30 cm long and 5 cm wide) with two opposing arms containing 30 cm high opaque walls. The light intensity used in the EPM was 15 ~ 20 Lux. Two mice were simultaneously placed in separate fields for the experiment. Each mouse was placed in a closed arm, facing the enter platform and cage mates started in the same closed arm with two overhead video tracking system for simultaneously tracing two independent mice movement and recording. Each mouse was allowed 5 min to explore the EPM and then returned to its home cage. Between tests the EPM was cleaned thoroughly with 70% alcohol. Open and closed arm entries were defined as the front two paws entering the arm, and open arm time began the moment the front paws entered the open arm and ended upon exit.

Microbiota analysis

16S rRNA sequencing

DNA extractions

DNA from different samples was extracted using the E.Z.N.A. ®Stool DNA Kit (D4015, Omega, Inc., USA) according to manufacturer’s instructions. The reagent which was

designed to uncover DNA from trace amounts of sample has been shown to be effective for the preparation of DNA of most bacteria. Nuclear-free water was used for blank. The total DNA was eluted in 50 μL of Elution buffer and stored at -80 °C until measurement in the PCR by LC-Bio Technology Co., Ltd, Hang Zhou, Zhejiang Province, China.

PCR amplification and 16S rRNA sequencing

The V3-V4 region of the prokaryotic (bacterial and archaeal) small-subunit (16S) rRNA gene was amplified with primers 341F (5'-CCTACGGGNGGCWGCAG-3') and 805R (5'-GACTACHVGGGTATCTAATCC-3') (1). The 5' ends of the primers were tagged with specific barcods per sample and sequencing universal primers. PCR amplification was performed in a total volume of 25 μL reaction mixture containing 25 ng of template DNA, 12.5 μL PCR Premix, 2.5 μL of each primer, and PCR-grade water to adjust the volume. The PCR conditions to amplify the prokaryotic 16S fragments consisted of an initial denaturation at 98 ℃ for 30 seconds; 32 cycles of denaturation at 98 ℃ for 10 seconds, annealing at 54 ℃ for 30 seconds, and extension at 72 ℃ for 45 seconds; and then final extension at 72 ℃ for 10 minutes. The PCR products were confirmed with 2% agarose gel electrophoresis. Throughout the DNA extraction process, ultrapure water, instead of a sample solution, was used to exclude the possibility of false-positive PCR results as a negative control. The PCR products were purified by AMPure XT beads (Beckman Coulter Genomics, Danvers, MA, USA) and quantified by Qubit (Invitrogen, USA). The amplicon pools were prepared for sequencing and the size and quantity of the amplicon library were assessed on Agilent 2100 Bioanalyzer (Agilent, USA) and with the Library Quantification Kit for Illumina (Kapa Biosciences, Woburn, MA, USA), respectively. The libraries were sequenced on NovaSeq PE250 platform.

Data analysis

Samples were sequenced on an Illumina NovaSeq platform according to the manufacturer's recommendations, provided by LC-Bio. Paired-end reads was assigned to samples based on their unique barcode and truncated by cutting off the barcode and primer sequence. Paired-end reads were merged using FLASH. Quality filtering on the raw reads were performed under specific filtering conditions to obtain the high-quality clean tags according to the fqtrim (v0.94). Chimeric sequences were filtered using Vsearch software (v2.3.4). After dereplication using DADA2, we obtained feature table and feature sequence. Alpha diversity and beta diversity were calculated by QIIME2, which of the same number of sequences were extracted randomly through reducing the number of sequences to the minimum of some samples, and the relative abundance (X bacteria count/total count) is used in bacteria taxonomy. Alpha diversity and Beta diversity were analyzed by QIIME2 process, and pictures were drawn by R (v3.5.2). The sequence alignment of species annotation was performed by QIIME2 plugin feature-classifier, and the alignment database was SILVA and NT-16S. Analysis of similarities (ANOSIM), redundancy analysis (RDA), and variation partitioning analysis (VPA) were carried out in the R package vegan (2). Linear discriminant analysis (LDA) effect size (LEfSe) and the Kruskal-Wallis (KW) rank sum test was performed to determine the differences in the abundances of taxa at the genus levels between the samples based on p < 0.05 and an LDA score >3.0 (3).

Sequences are available on Sequence Read Archive, BioProject ID PRJNA804655.

Results

**Comparison of** **microbial diversity of intestine from childhood to adulthood**

The rarefaction curves of good coverage and observed outs showed that curves had reached a flat level, suggesting that the sequencing depth was sufficient (Supplementary Figure 1A, B). There was neither significant difference in alpha (α) diversity of ileal contents of microbiota between MS and NS at PND 30 nor significant difference at PND 75, respectively (*P* > 0.05**)** (supplementary Figure 1C, 1D). However, ELS significantly affect beta (β) diversity of ileal contents of microbiota at PND 30 and PND 75 (supplementary Figure 1E, 1F). In addition, there was neither significant difference in α diversity of fecal samples of microbiota between MS and NS at PND 30, PND 45, and PND 75, respectively (*P* > 0.05) (supplementary Figure 1C, 1D) nor significant difference in β diversity of fecal samples of microbiota between MS and NS at PND 30, PND 45, and PND 75, respectively (*P* > 0.05) (supplementary Figure 1E, 1F).


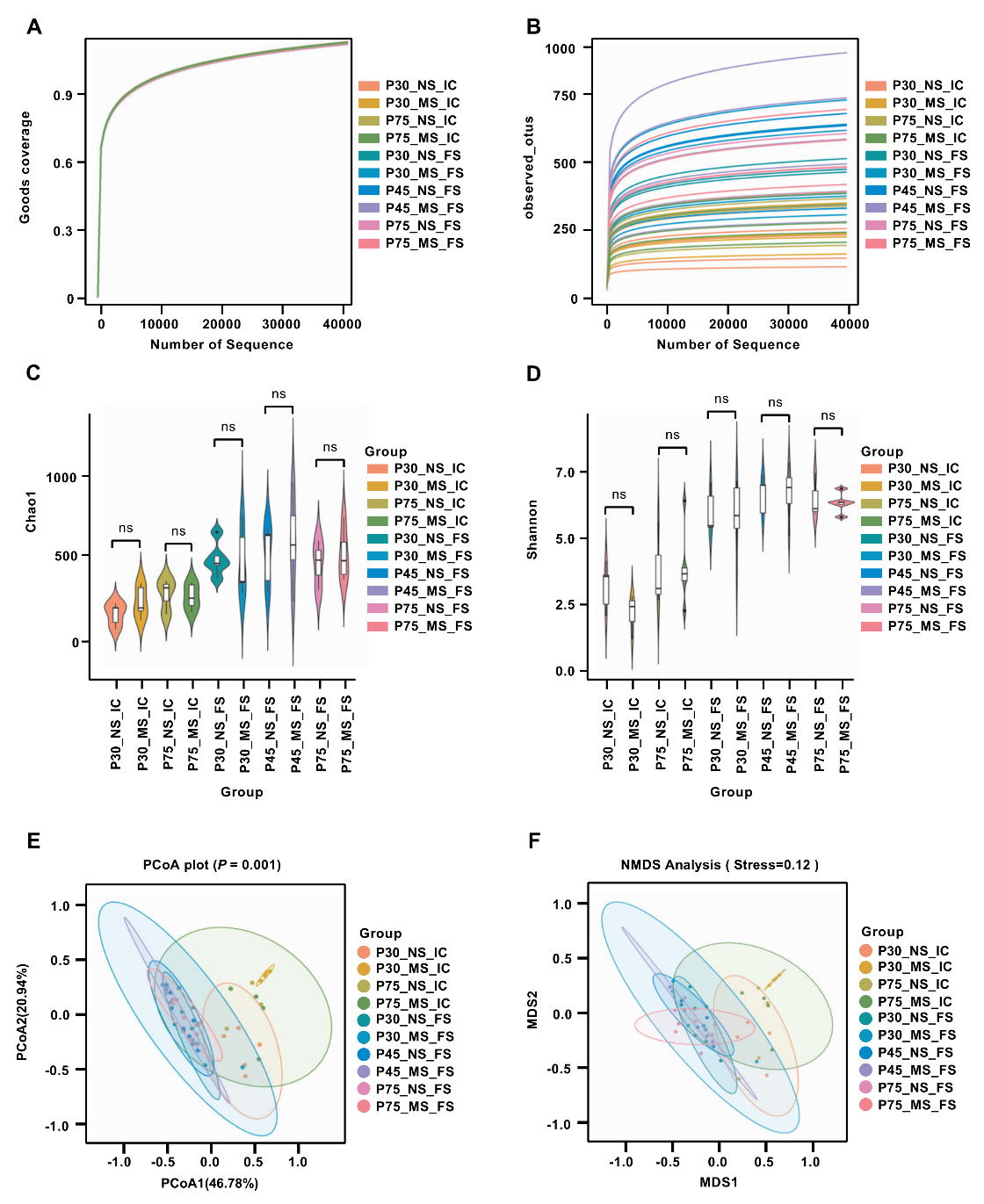


Figure S1 Impact of early life stress on microbial diversity of intestine from childhood to adulthood. (A, B): Evaluation of sequencing depth of gut microbiota with goods coverage and observed otus; (C, D): Comparison of alpha diversity, chao 1 and Shannon index, respectively; (E): Principal coordinates analysis of gut microbiota between MS and NS. *P* value were calculated using ANOSIM. (F): NMDS analysis of gut microbiota between maternal separation (MS) and non-separation (NS).

**Impact of ELS on microbial composition of ileal contents at phylum level**


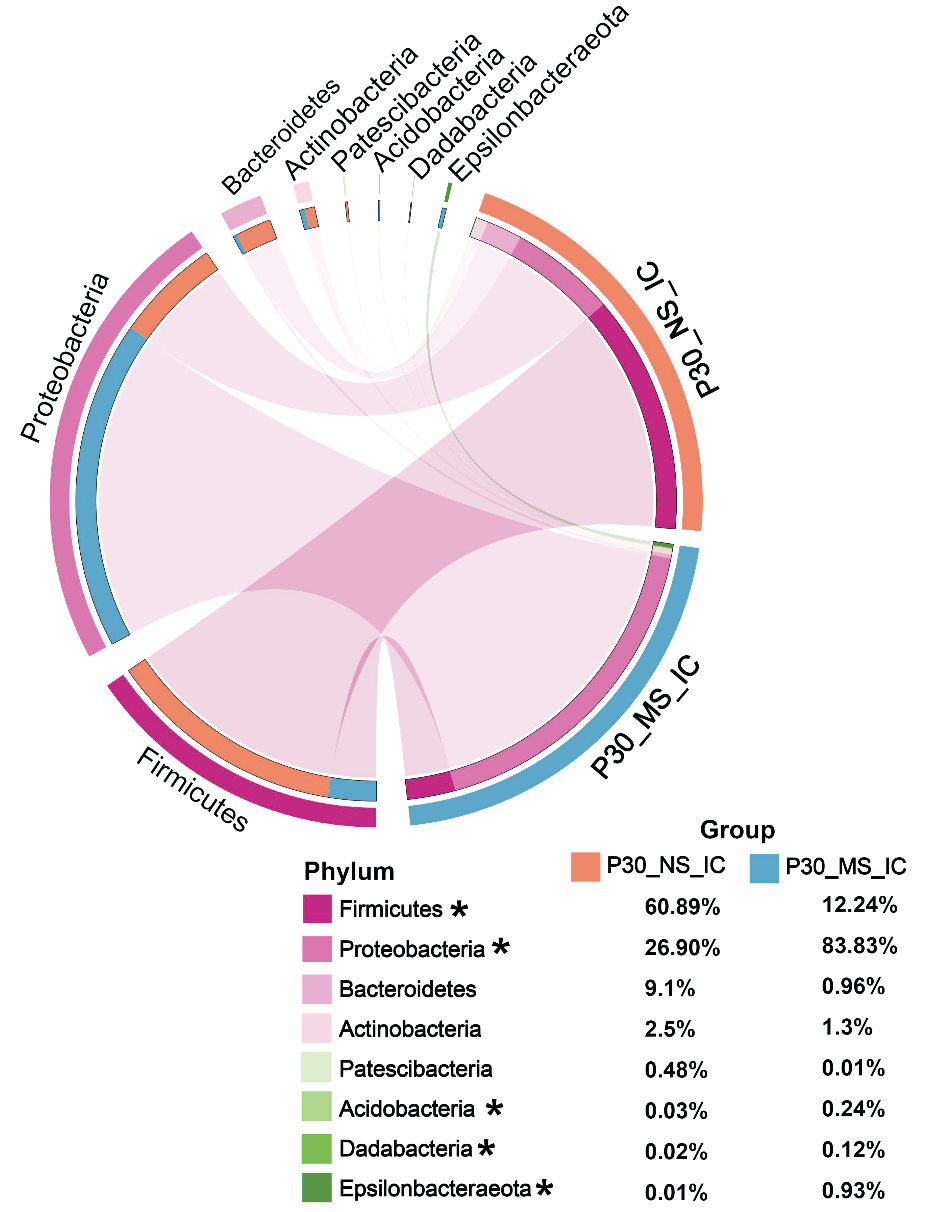
ELS significantly affect microbiota community of ileal contents at PND 30. Concerning the phylum level, the microbiota of the MS group was characterized by significantly high levels of *Proteobacteria*, *Epsilonbacteraeota, Acidobacteria,* and *Dadabacteria,* and low level of *Firmicutes* in comparison with the NS group (Supplementary Figure 2).

Figure S2 Impact of early life stress on microbial composition of ileal contents at Phylum level at postnatal day 30. *: *P* < 0.05.

In addition, ELS significantly affect microbiota community of ileal contents at PND 75. The abundance of *Cyanobacteria* was significantly lower in the MS group in comparison of the NS group, but the composition of the four mains of phylum, such as *Proteobacteria, Firmicutes, Actinobacteria,* and *Bacteroidetes* had no significant difference. (Supplementary Figure 3).

Figure S3 Impact of early life stress on microbial composition of ileal contents at Phylum level at postnatal day 75. *: *P* < 0.05.


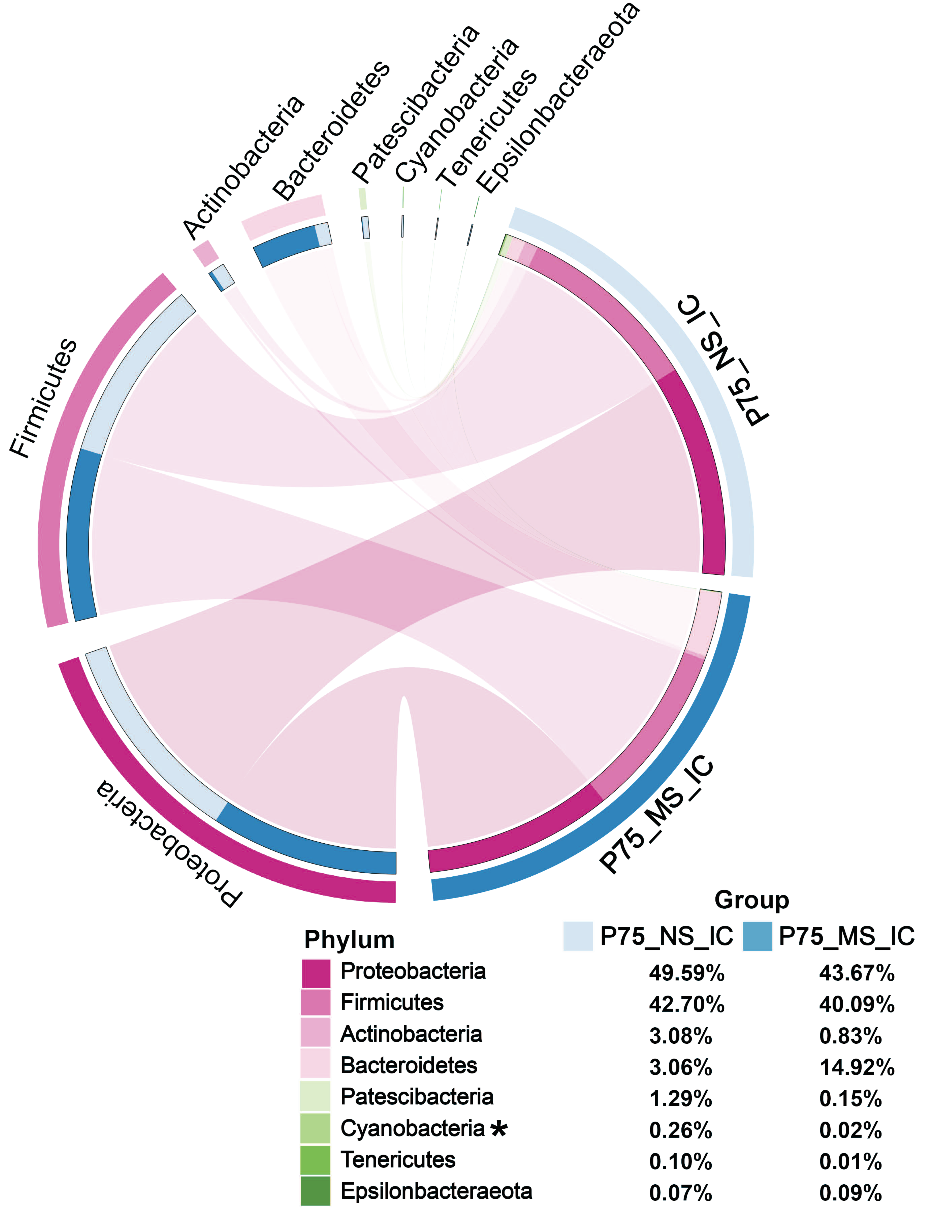
For the specific microbiota community differences of ileal contents at various taxonomic levels caused by ELS between MS and NS at PND 30 (supplementary Figure 4, 5), and PND 75 (supplementary Figure 6) please refer to linear discriminant analysis effect size (LEfSe) and linear discriminant analysis (LDA) score.


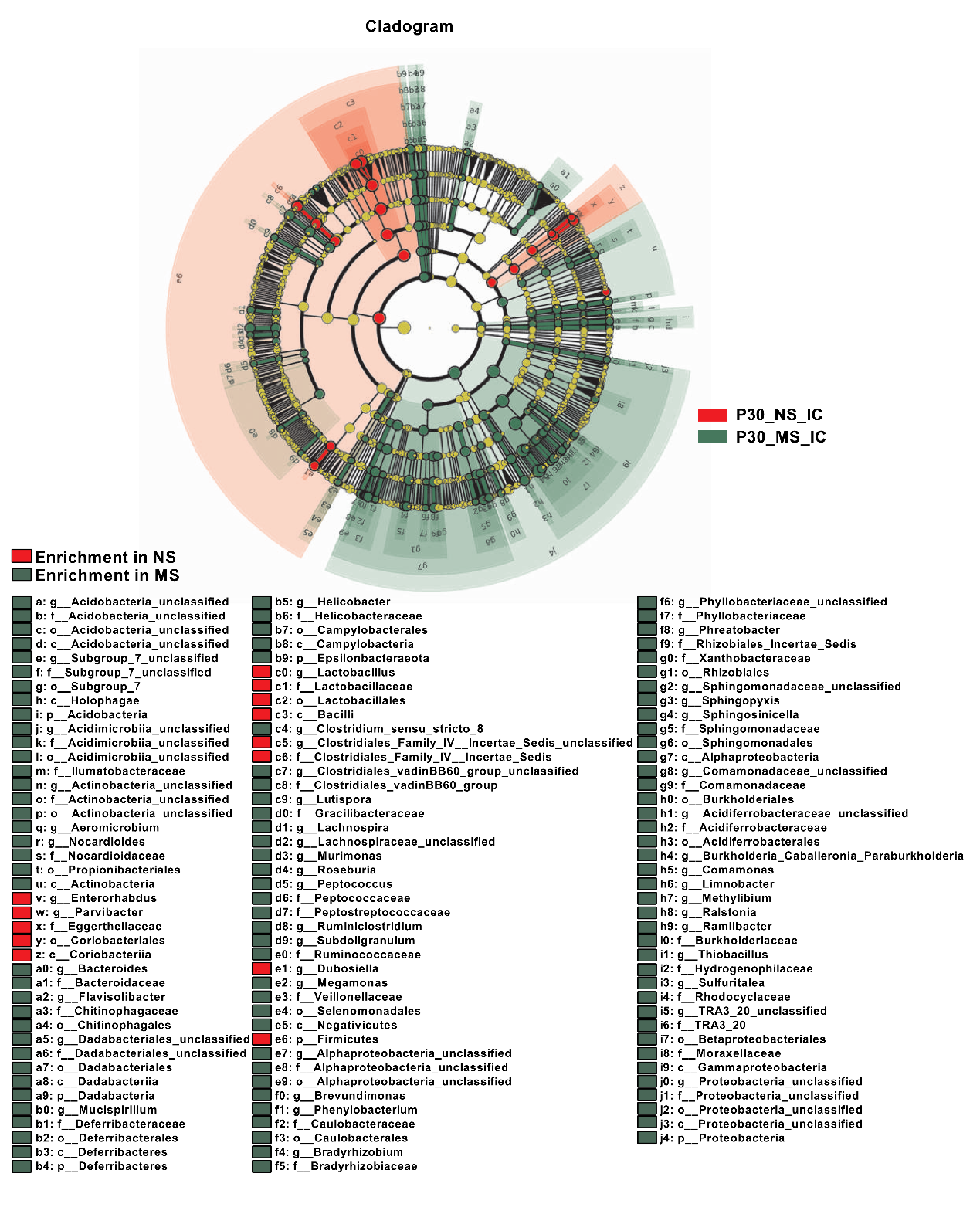


Figure S4 LEfSe displays the impact of early life stress on specific microbiota community differences at various taxonomic levels at postnatal day 30.


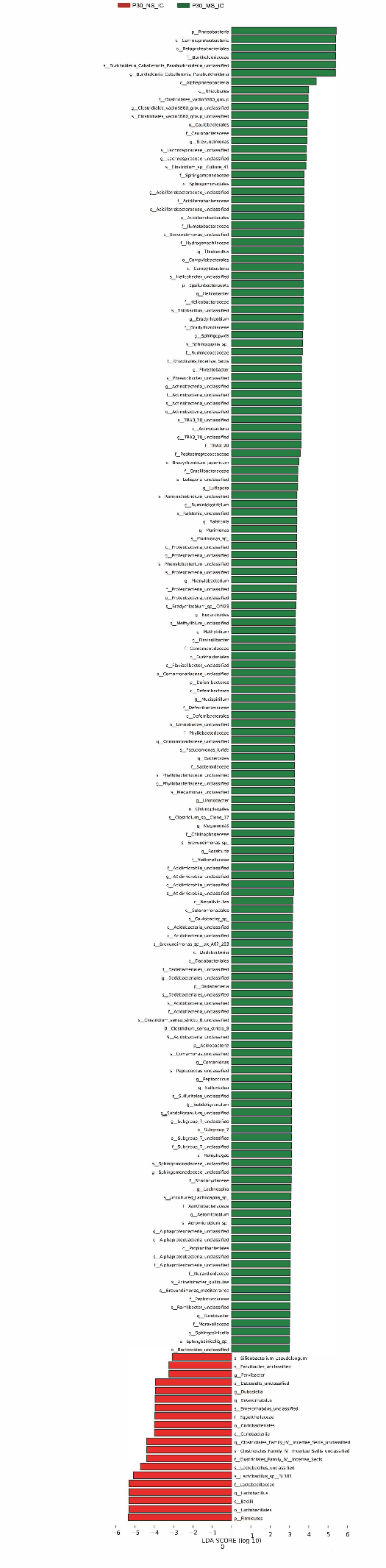


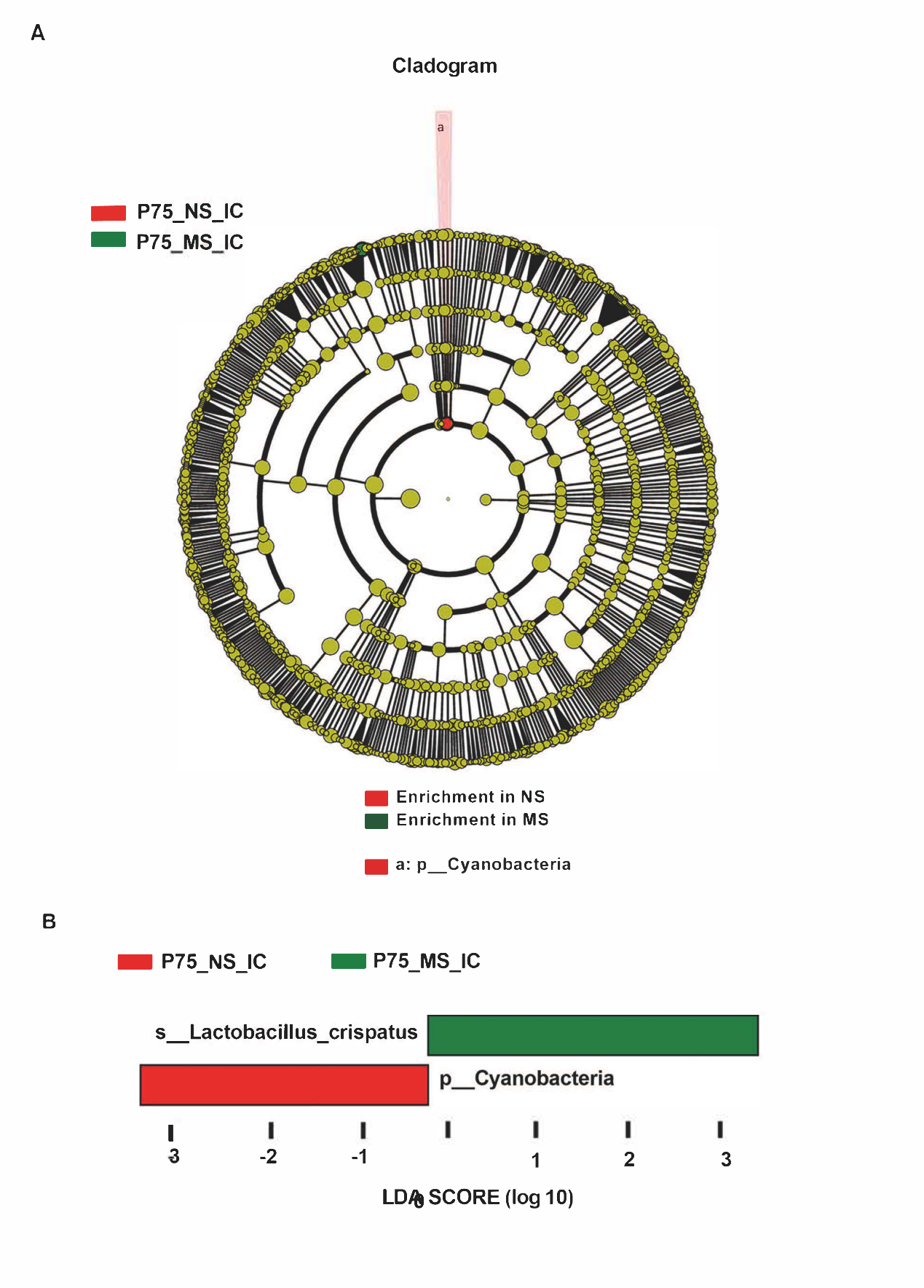


Figure S5 LDA score of Lefse-PICRUSt displays the differences at various taxonomic levels caused by early life stress between MS and NS at postnatal day 30.

**Impact of ELS on microbial composition of fecal samples at phylum level**

Figure S6 LEfSe integrated with LDA score comparison of relative abundance of ileal contents microbiota between maternal separation and non-separation group at postnatal day 75. (A): Lefse analysis. (B): LDA Effect Size.

At phylum level, although ELS did not affect microbial composition of fecal samples

at PND 30 (supplementary Figure 7) and PND 75 (supplementary Figure 9), it significantly affected microbial composition of fecal samples at PND 45 (supplementary Figure 8). The abundance of *Patescibacteria* phylum was observed significantly more enriched in the MS group than that in the NS group at PND 45. Conversely, *Tenericutes* and *Cyanobacteria* phylum was found lower relative abundance in the MS group than that in the NS group at PND 45 (supplementary Figure 8).


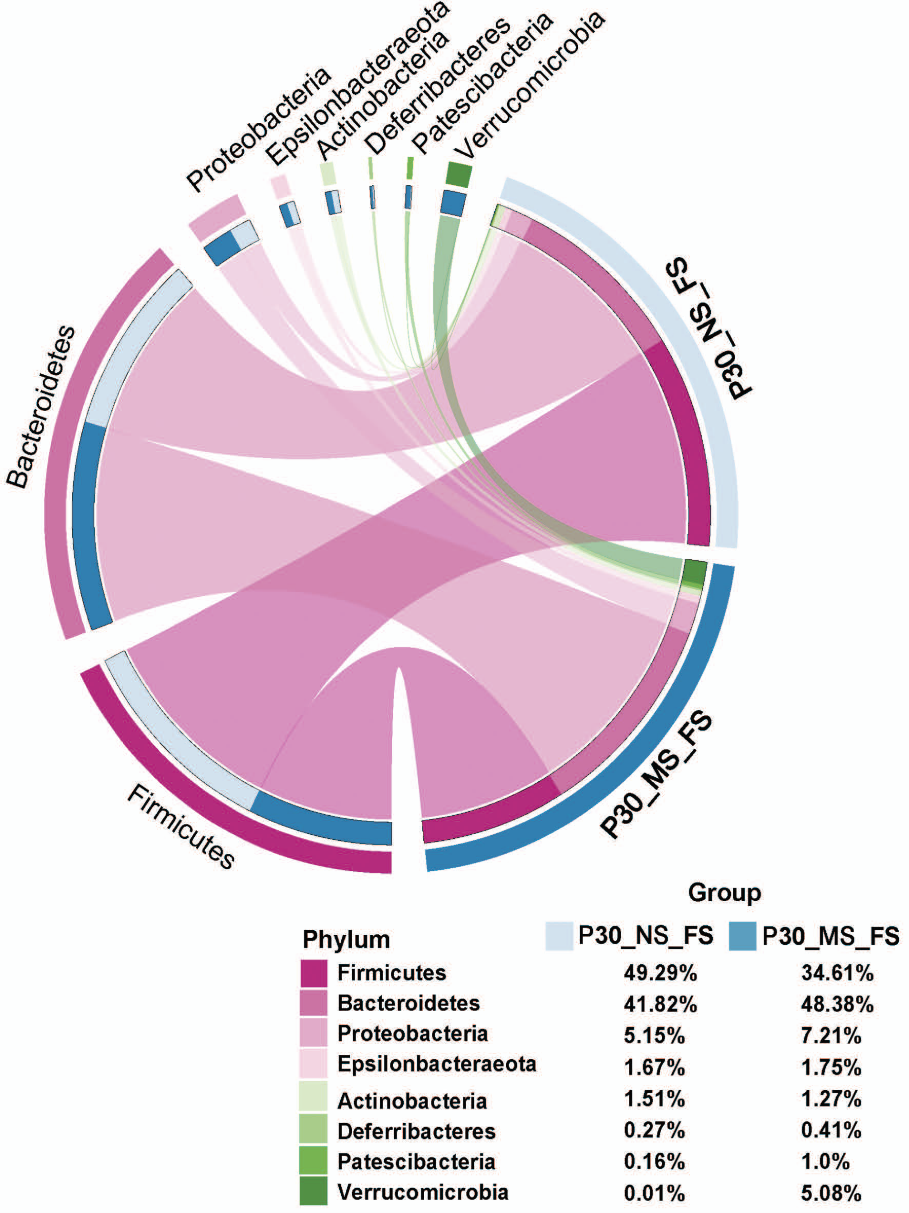


Figure S7 Impact of early life stress on microbial composition of fecal samples at Phylum level at postnatal day 30.


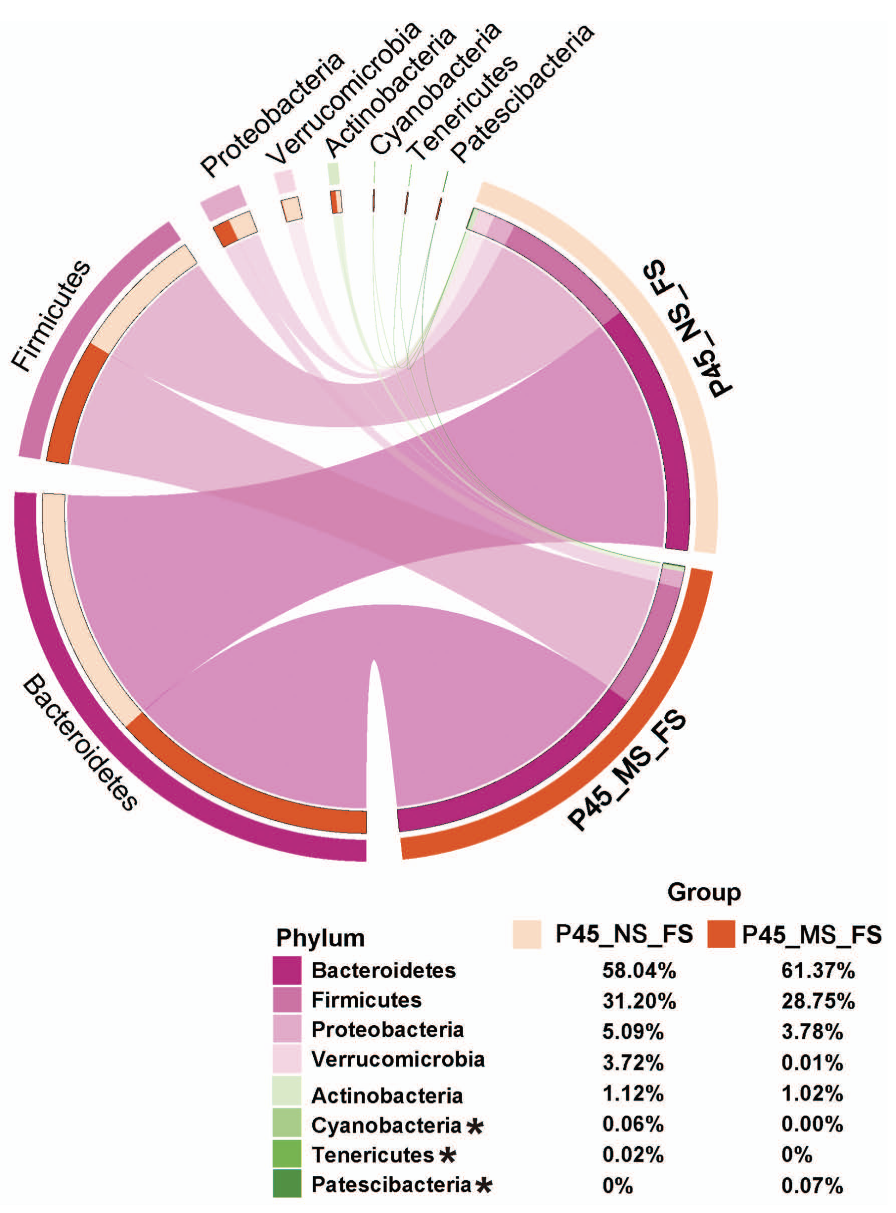


Figure S8 Impact of early life stress on microbial composition of fecal samples at Phylum level at postnatal day 45. *: *P* < 0.05.


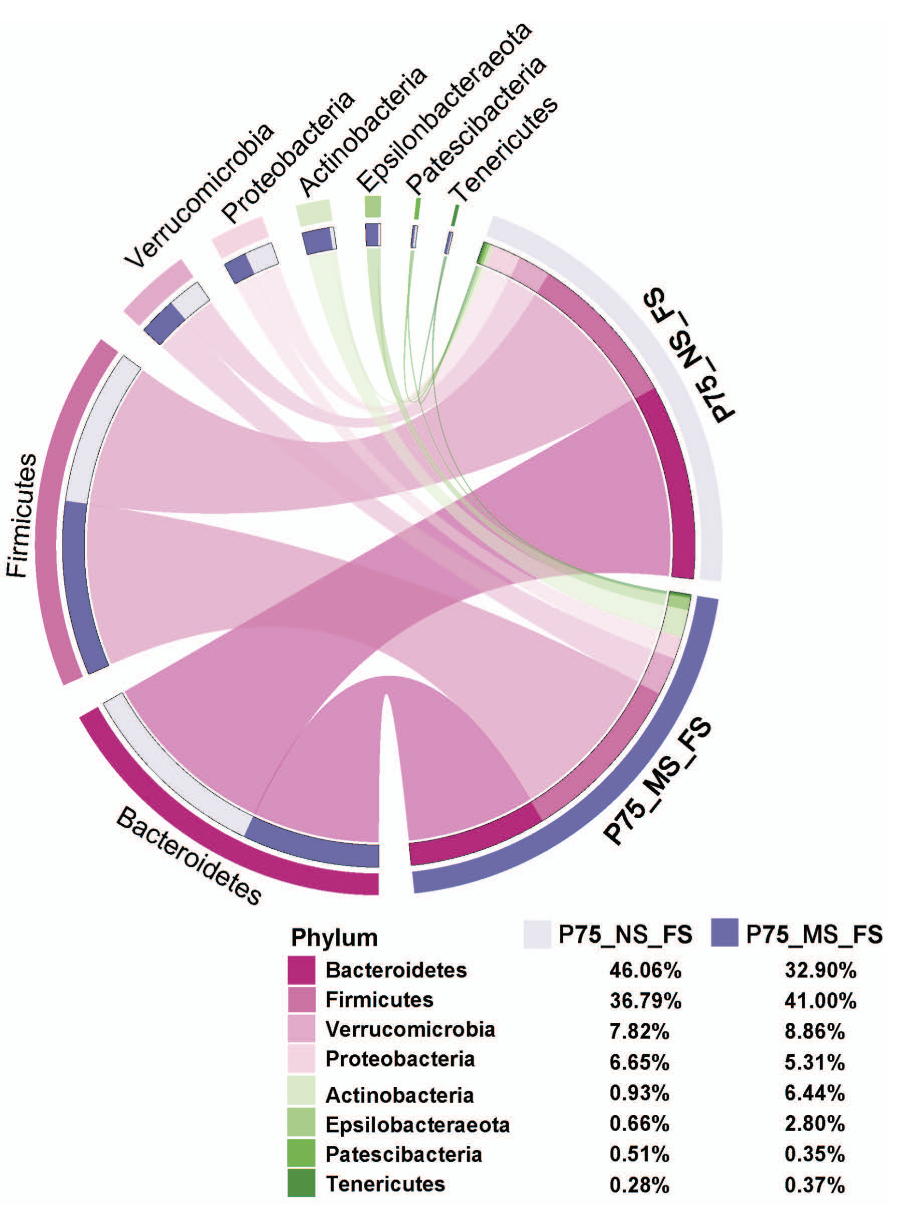


Figure S9 Impact of early life stress on microbial composition of fecal samples at Phylum level at postnatal day 75.

For the specific microbiota community differences of fecal samples at various taxonomic levels caused by ELS between MS and NS at PND 30 (Supplementary figure 10), PND 45 (Supplementary figure 11) and PND 75 (Supplementary figure 12) please refer to LEfSe, and LDA score.


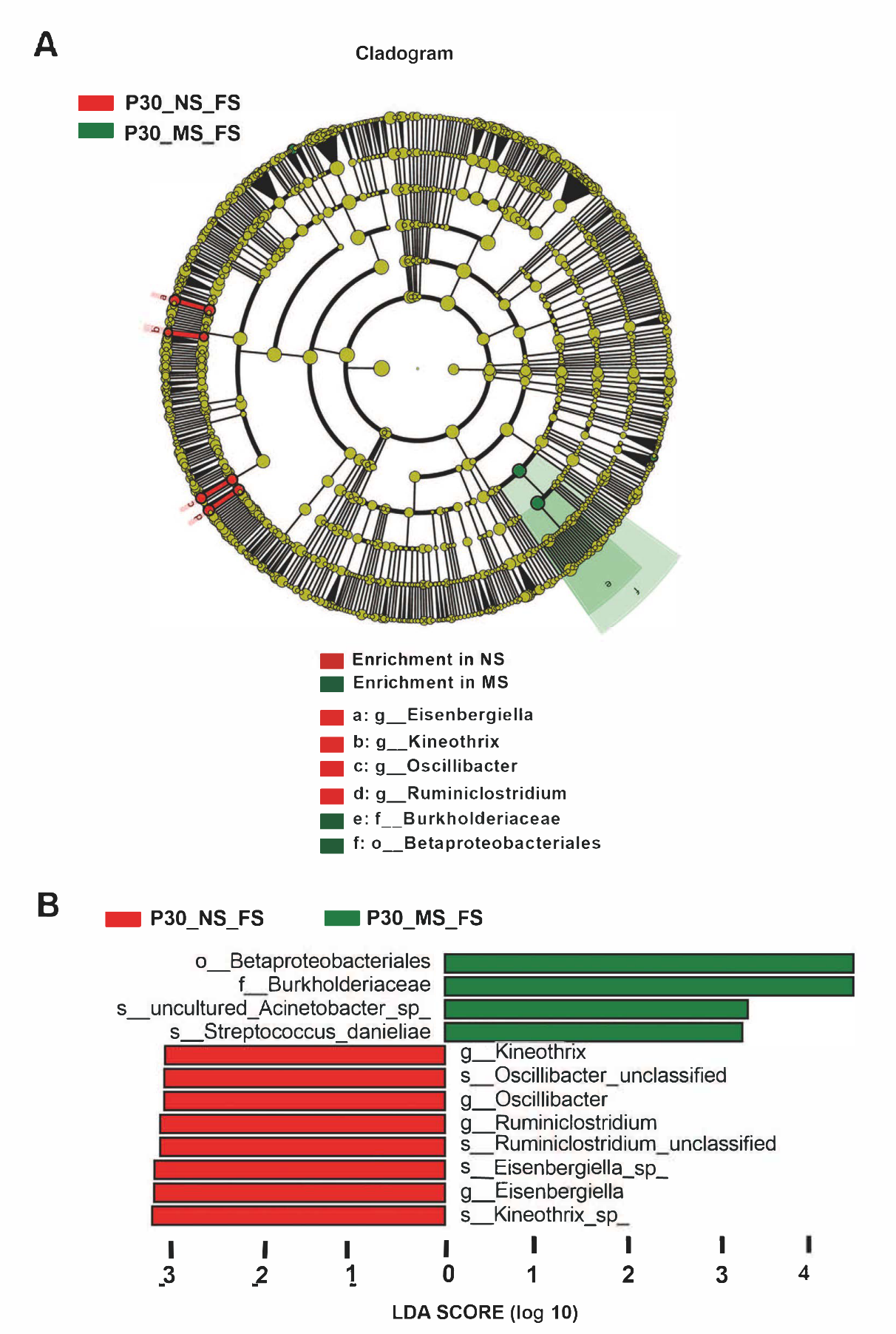


Figure S10 LEfSe integrated with LDA score comparison of relative abundance of fecal samples microbiota between maternal separation and non-separation group at postnatal day 30. (A): Lefse analysis. (B): LDA Effect Size.


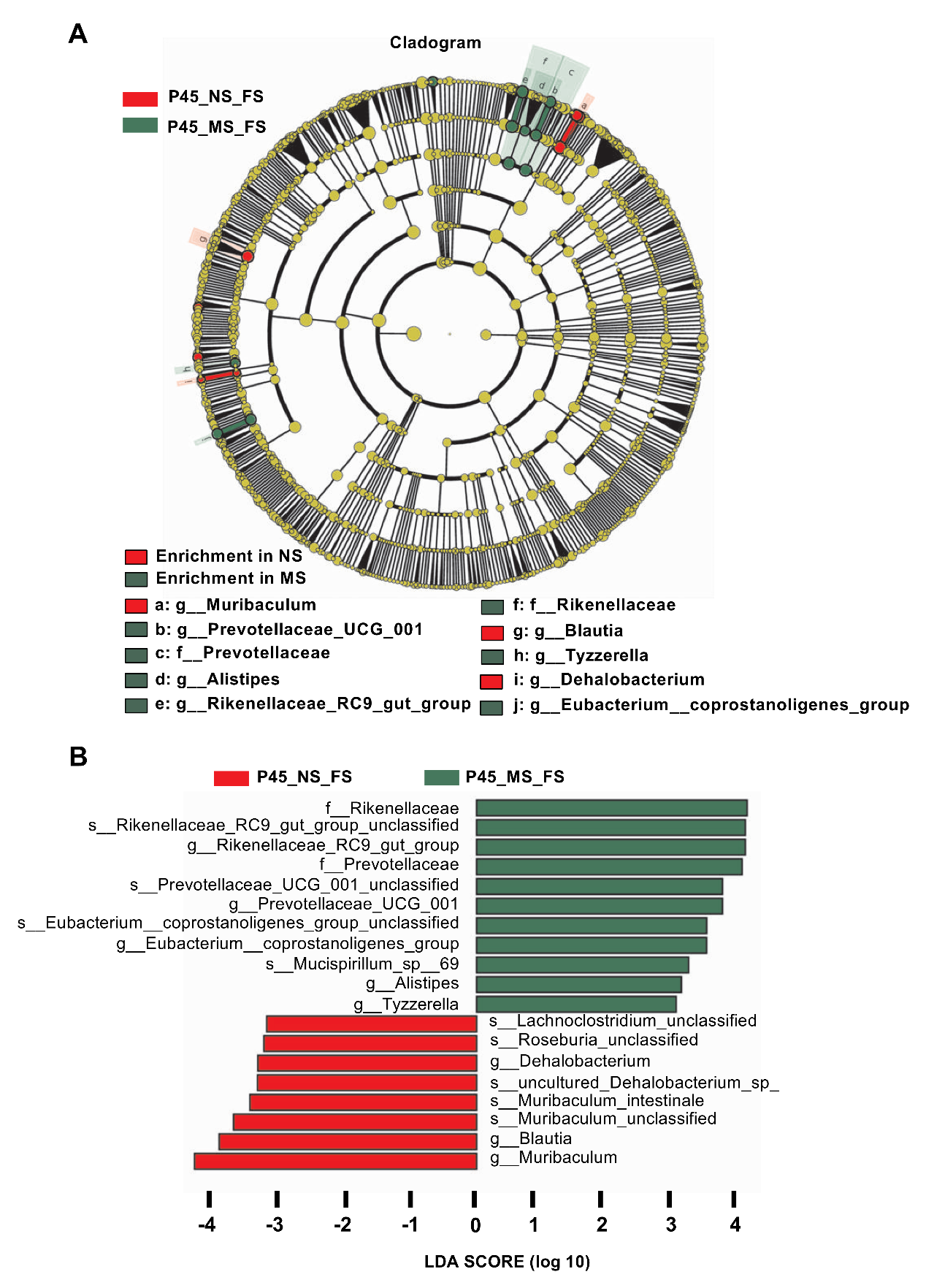


Figure S11 LEfSe integrated with LDA score comparison of relative abundance of fecal samples microbiota between maternal separation and non-separation group at postnatal day 45. (A): Lefse analysis. (B): LDA Effect Size.


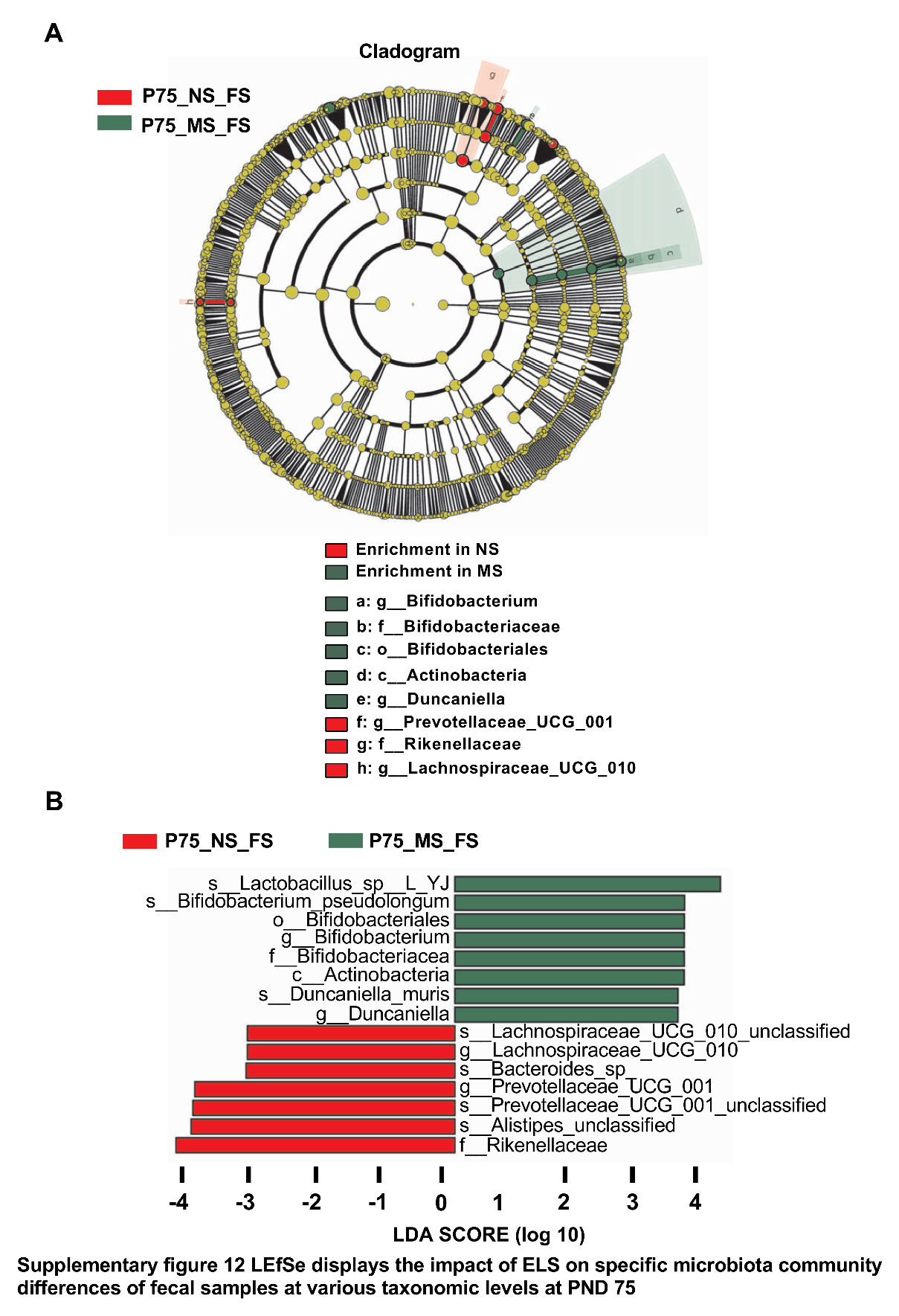


Figure S12 LEfSe integrated with LDA score comparison of relative abundance of fecal samples microbiota between maternal separation and non-separation group at postnatal day 75. (A): Lefse analysis. (B): LDA Effect Size.

References

1. Logue JB, Stedmon CA, Kellerman AM, et al. Experimental insights into the importance of aquatic bacterial community composition to the degradation of dissolved organic matter. Isme j 2016;10:533-45.

2. J. Oksanen, F. Blanchet, M. Friendly, R. Kindt, P. Legendre, D. McGlinn, P. Minchin, R. O’Hara, G. Simpson, P. Solymos, vegan: Community Ecology Package (R package version 2.5–5), in, 2019.

3. Segata N, Izard J, Waldron L, et al. Metagenomic biomarker discovery and explanation. Genome Biol 2011;12:R60.
